# Supplementary material for: An Essential Role for Liver ERα in Coupling Hepatic Metabolism to the Reproductive Cycle
Source: Cell Rep. 2016 Mar 31;15(2):360–71. doi: 10.1016/j.celrep.2016.03.019 (PMC4835581; doi:10.1016/j.celrep.2016.03.019)
Supplement: Document S1. Supplemental Experimental Procedures, Figures S1–S7, and Table S1 [file mmc1.pdf]

Cell Reports, Volume *15*

Supplemental Information

An Essential Role for Liver ERα in Coupling Hepatic Metabolism to the Reproductive Cycle

Sara Della Torre, Nico Mitro, Roberta Fontana, Monica Gomaraschi, Elda Favari, Camilla Recordati, Federica Lolli, Fabiana Quagliarini, Clara Meda, Claes Ohlsson, Maurizio Crestani, Nina Henriette Uhlenhaut, Laura Calabresi, and Adriana Maggi

Figure S1, related to Figure 1

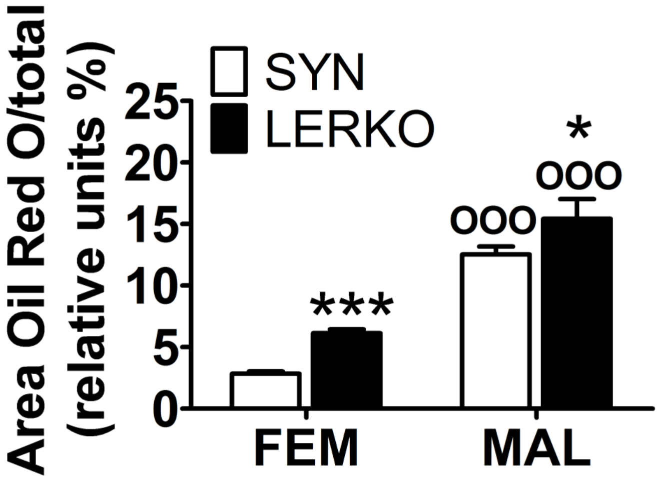

Figure S1 (related to Figure 1). Liver fat deposition in SYN and LERKO female and male mice.

Quantification of the lipid deposits by Oil Red O staining in the liver tissues of SYN and LERKO female (FEM) and male (MAL) mice of 10 months of age. The data are expressed as percentages of the total section areas. The bars are mean ± SEM, n=6; the experiment was repeated 3 times. **p<0.01 and ***p<0.001 *versus* SYN; ^{OOO}p<0.001 *versus* females by two-way ANOVA followed by Bonferroni *post-hoc* test.

Figure S2, related to Figure 1

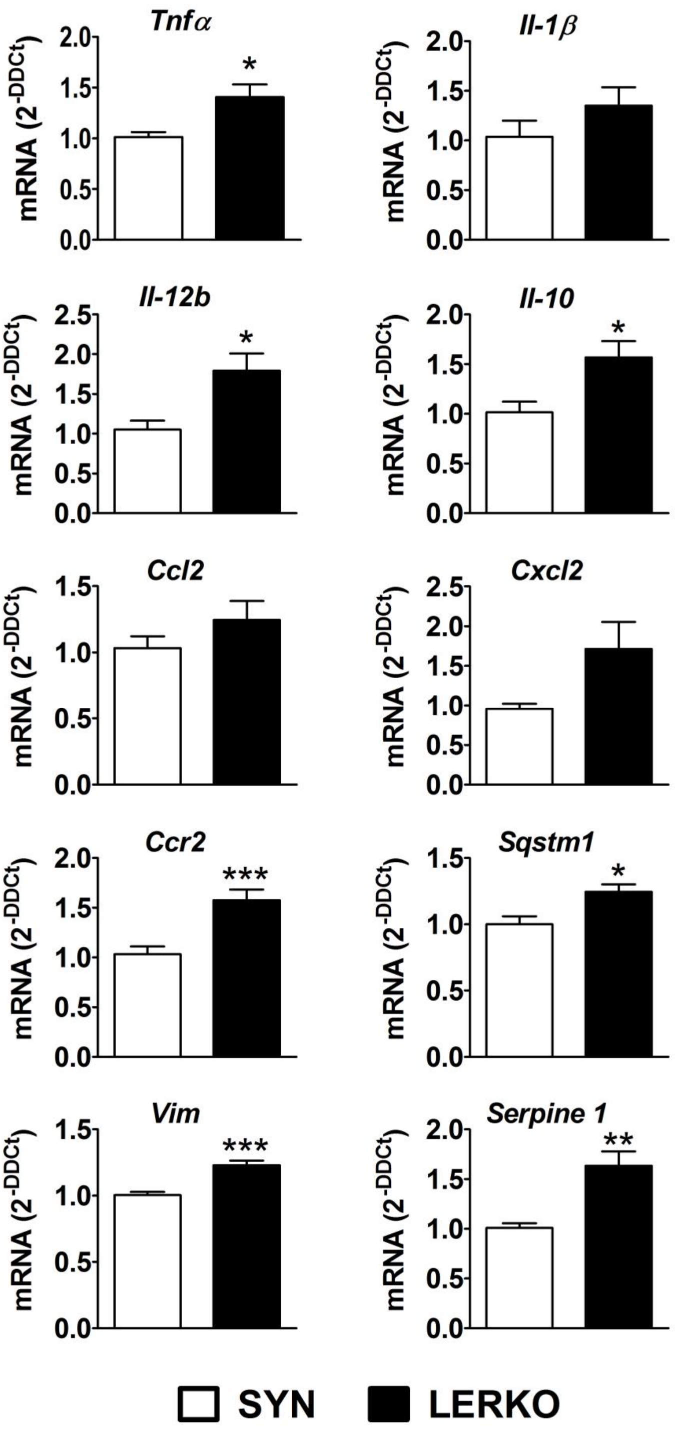

Figure S2 (related to Figure 1). **The contents of inflammatory marker mRNAs in the liver tissues of SYN and LERKO females (at metestrus) at 10 months of age**. The expression of several genes involved in the inflammatory process (tumor necrosis factor alpha, *TnfŬ*; interleukin-1 beta, *Il-1ɓ*; interleukin-12 beta; *Il-12ɓ*; interleukin-10, *Il-10*; chemokine (C-C motif) ligand 2, *Ccl2*; chemokine (C-X-C motif) ligand 2, *Cxcl2*; chemokine (C-C motif) receptor 2, *Ccr2*) and collagen deposition (sequestosome1, *Sqstm1*; vimentin, *Vim*; serpine1, *Serpine*) was measured by rtPCR. The bars are mean ± SEM, n=15. *p<0.05 and **p<0.01 *versus* SYN by t-test.

Figure S3, related to Figure 2

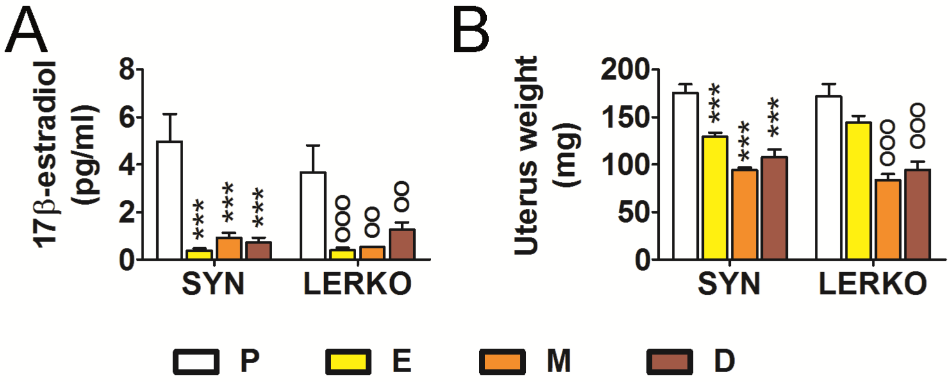

Figure S3 (related to Figure 2). Circulating 17β-estradiol levels and uterine weight changes in SYN and LERKO females during the estrous cycle progression. A. 17β-estradiol levels measured in the plasma of SYN and LERKO females during the estrous cycle. The bars are mean ± SEM, n=4. ***p<0.001 *versus* SYN at P; ^{OO}p<0.01 and ^{OOO}p<0.001 *versus* LERKO at P by two-way ANOVA followed by Bonferroni *post-hoc* test. **B.** Uterine weight changes in SYN and LERKO females during the estrous cycle progression. The bars are mean ± SEM, n=6; the experiment was repeated 3 times. ***p<0.001 *versus* SYN at P; ^{OOO}p<0.001 *versus* LERKO at P by two-way ANOVA followed by Bonferroni *post-hoc* test.

Figure S4, related to Figure 3

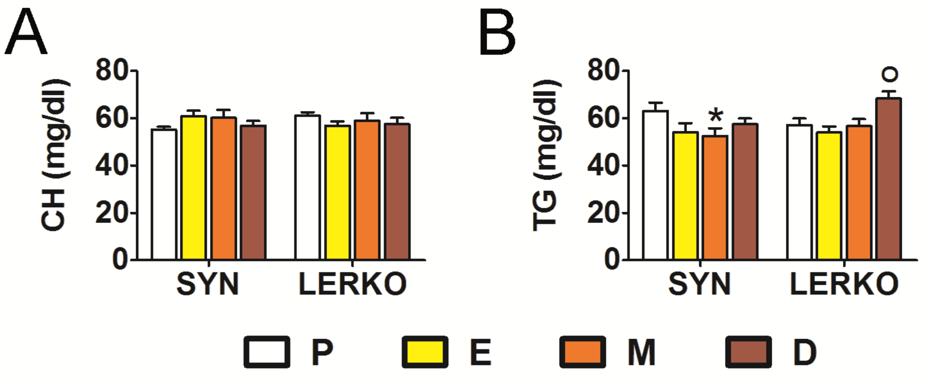

Figure S4 (related to Figure 3). Total cholesterol and triglycerides in the plasmas of SYN and LERKO females during the estrous cycle. The bars are mean ± SEM, n=6; the experiment was repeated 3 times. *p<0.05 *versus* SYN at P; ^{O}p<0.05 *versus* LERKO at P by two-way ANOVA followed by Bonferroni *post-hoc* test.

Figure S5, related to Figure 3A

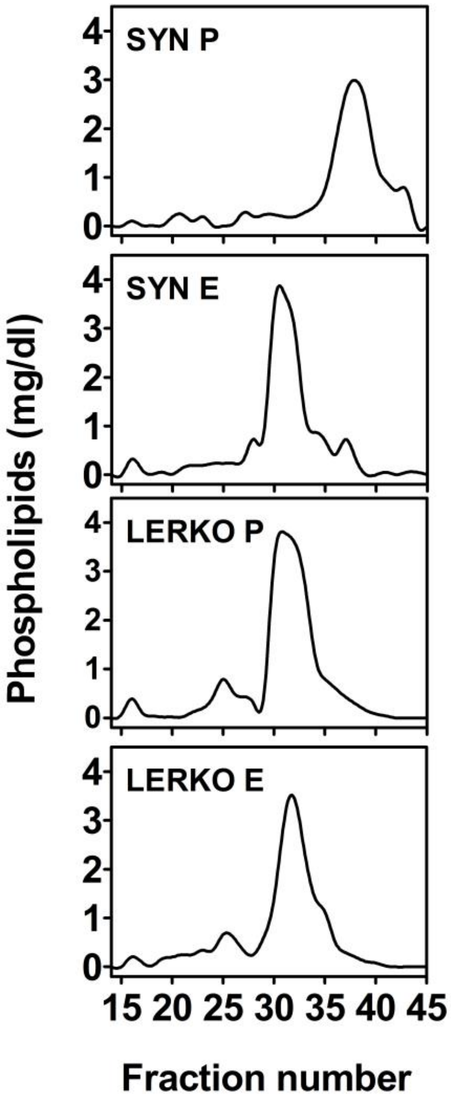

Figure S5 (related to Figure 3A). **Phospholipid profiles of the FPLC-fractioned plasma samples from the SYN and LERKO females.** The graph shows the phospholipid contents in mg/dl that were measured in FPLC-fractioned plasmas of the SYN and LERKO females at proestrus (P) and estrus (E).

Figure S6, related to Figure 3

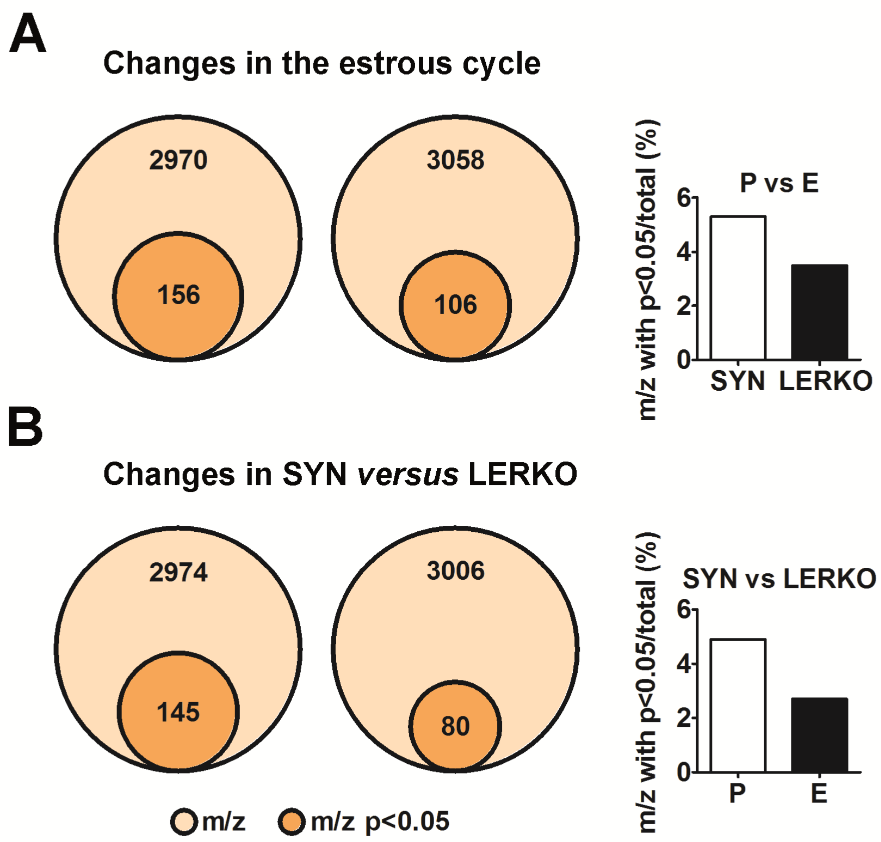

Figure S6 (related to Figure 3). Plasma metabolomic analyses of the SYN and LERKO females. A. The pie charts represent the total number of metabolites (light orange) and the number of metabolites that were differentially expressed (dark orange) between P and E in the SYN and LERKO mice. The graph represents the number of metabolites differentially modulated between P and E expressed as a percentage respect the total number of metabolites that were identified in the plasmas of the SYN and LERKO females. **B**. The pie chart represents the total number of metabolites (light orange) and the number of metabolites that were differentially expressed (dark orange) at P and E in the SYN and LERKO mice. The graph represents the number of metabolites that were differentially regulated between the SYN and LERKO mice expressed as percentages of the total number of metabolites identified in the plasmas of the females at P and E, respectively.

Figure S7, related to Figure 6

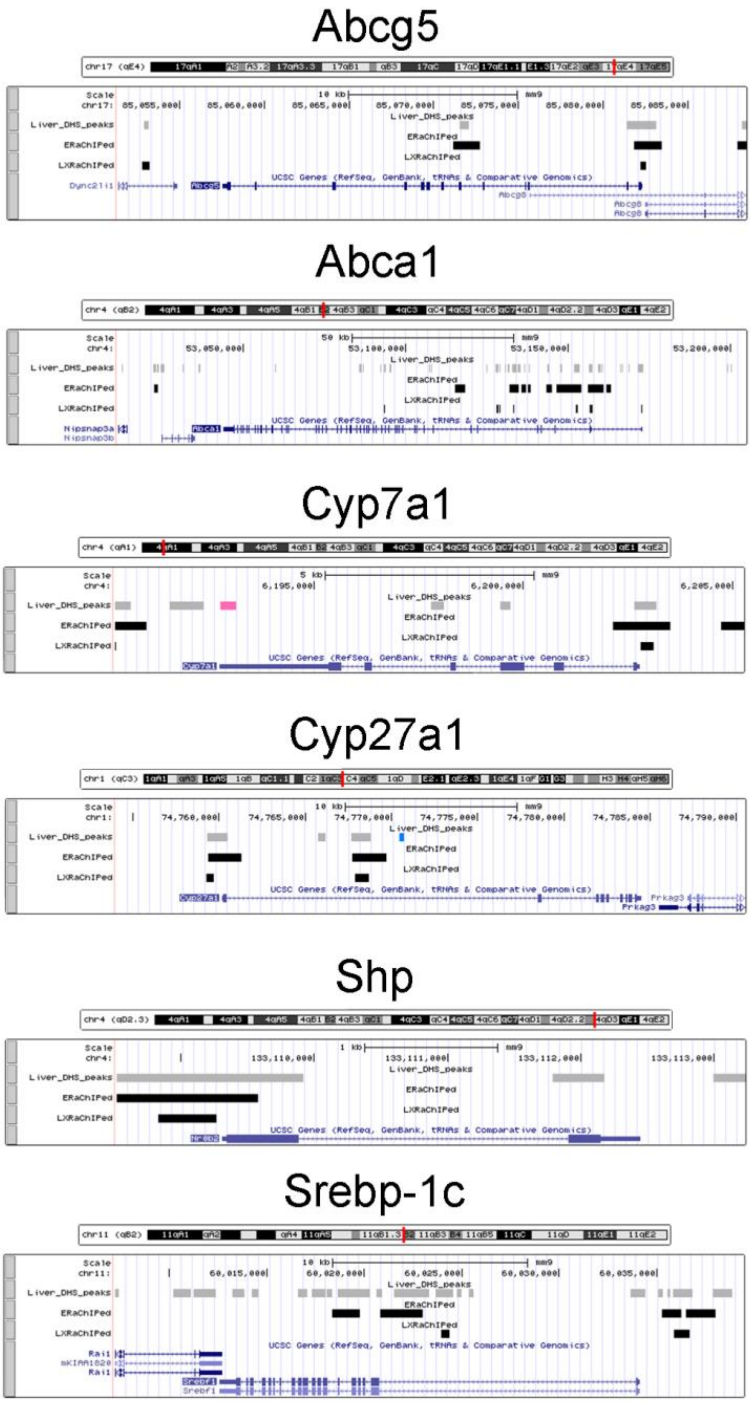

Figure S7 (related to Figure 6). **UCSC Genome Browser tracks derived from ChIP-seq data showing ERŬ and LXRŬ binding to the *Abcg5*, *Abca1*, *Cyp7a1*, *Cyp27a1*, *Shp*, and *Srebp-1c***. The black bars indicate binding of ERŬ (labeled as ñUser Track: ERŬChIPedò) and LXRŬ (labeled as ñUser Track: LXRŬChIPedò). The gray bars show the liver DHS peaks identified by analyzing the genome-wide DNase hypersensitivity in mouse liver mapped by DNase I treatment coupled with high-throughput sequencing (DNase-seq) (Rozowsky et al., 2009).

Table S1

	Females SYN	Females LERKO
Hepatocellular vacuolar degeneration (fatty change)	++	++
Droplet size	+	++/+++
Portal inflammation	+	++
Inflammatory foci	-	+
Portal fibrosis	-	-
Portal and/or centrilobular fibrosis	-	+/++

Table S1. **Anatomo-pathological analysis of the livers of SYN and LERKO female mice at 10 months of age.** Liver sections were routinely stained with hematoxylin and eosin (H&E) and evaluated in a blinded fashion under a light microscope. A semiquantitative grading of hepatocellular vacuolar degeneration and portal inflammation (i.e., the infiltration of mononuclear inflammatory cells) was performed.

SUPPLEMENTAL EXPERIMENTAL PROCEDURES

Liver histology: The left lobe of the liver was fixed in 10% neutral formalin solution (Sigma-Aldrich) overnight at 4°C, cryopreserved in a 30% (w/v) sucrose solution for 24 hours at 4°C and stored at -80°C. 7 µm-thick liver sections were cut with a refrigerated microtome (Leica), collected on slides and stored at -80°C until staining. Hematoxylinï eosin (H&E) staining was performed on the frozen slides with Mayer hematoxylin (Bio-Optica) for 1 min and, after washing with water, with 1% eosin aqueous solution (Bio-Optica) for 4 minutes. Oil Red O staining was performed as previously described (Villa et al., 2012). The Accustain Trichrome stain kit was used for Massonôs trichrome staining (Sigma-Aldrich). After staining, the slides were cleared in xylenes and cover slipped with xylenes-based mounting medium (Eukitt, Bio-Optica). The liver sections were evaluated in a blinded fashion under a light microscope. Semi-quantitative grading of the hepatocellular vacuolar degeneration and portal inflammation (i.e., the infiltration of mononuclear inflammatory cells) was performed in a blinded fashion. Images of the stained sections were captured using Microscope Axioscop2 mot plus (Zeiss).

Real-Time PCR Gene Expression Analysis: The primers used for the rtPCR reactions were as follows: *Esr1* (Mm00433147_m1), *Pltp* (Mm01240573_m1), *Lipc* (Mm01171487_m1), *PpparŬ* (Mm00440939_m1), *Cpt1Ŭ* (Mm01231183_m1), *HadhŬ* (Mm00805228_m1), *Acox* (Mm01246831_m1), *Acsl1* (Mm00484217_m1), *LxrŬ* (Mm00443451_m1), *Abcg55* (Mm00446241_m1), *Abca1* (Mm00442646_m1), *Cyp7a1* (Mm00484150_m1), *Cyp27a1* (Mm00470430_m1), *Fasn* (Mm00662319_m1), *TnfŬ* (Mm00443258_m1), *Il-1ɓ* (Mm00434228_m1), *Il-12ɓ* (Mm00434174_m1), *Il-10* (Mm00439614_m1), *Ccl2* (Mm00441242_m1), *Cxcl2* (Mm00436450_m1), *Ccr2* (Mm00438270_m1), *Sqstm1* (Mm00448091_m1), *Vim* (Mm01333430_m1), *Serpine* (Mm00435860_m1), *Shp* (Mm00442278_m1) (all from Life Technologies), and *Srebp-1c* (forward: 5´-ATGGATTGCACATTTGAAGACATGCT-3´ and reverse: 5´-CCTGTGTCCCCTGTCTCAC-3´). The *36b4* primer was been used as reference gene assay (forward: 5´-GGCGACCTGGAAGTCCAACT-3´ and reverse: 5´-CCATCAGCACCACAGCCTTC-3´).

17ɓ-estradiol levels in the plasma: Plasma levels of estradiol were measured in a single run by GC-MS/MS. Briefly, after addition of isotope-labeled standards, estradiol was extracted to chlorobutane, purified on a silica column and derivatized using pentafluorobenzyl hydroxylamine hydrochloride followed by pentafluorobenzoyl chloride. Estradiol was analyzed in multiple reaction monitoring mode with ammonia as reagent gas using an Agilent 7000 triple quadrupole mass spectrometer equipped with a chemical ionization source. The limits of quantification (LOQ) for estradiol was 0.5 pg/ml. The intra-assay coefficients of variation (CVs) were below 14% and the inter-assay CVs below 11%.

Western blot analysis: Samples of frozen mouse liver were homogenized in ice-cold buffer (20 mM HEPES, 5 mM $MgCl_2$, 420 mM NaCl, 0.1 mM EDTA, and 20% glycerol) containing protease and phosphatase inhibitors according to the manufacturerôs protocols (Phosphatase and Protease Inhibitor Mini Tablets, Pierce). After 3 repeated cycles of freezing and thawing, the homogenate was centrifuged at 16100g for 15 min at 4°C, and the supernatant was collected in a new tube. After appropriate quantitative analysis (Bradford assay, Pierce), equal amounts of the protein samples (25 µg of liver extracts or 30 µL of plasma FPLC fractions) were resuspended in Laemmli sample buffer and separated in an 8-16% SDS polyacrylamide gel system (Biorad). After transfer, the nitrocellulose membranes were incubated with specific antibodies overnight at 4°C and then with the secondary antibody conjugated with peroxidase for 1h at r.t. The primary antibodies used were the following: anti-apoAI (Rockland Immunochemicals), anti-apoE (Calbiochem), anti-LDLR and anti-SR-B1 (both Novus Biological), anti-ERŬ and anti-PPARŬ (Santa Cruz), anti-LXRŬ (R&D systems), and anti-ɓ-actin (Sigma). Immunoreactivity was detected with an ECL Western Blotting Analysis System (Amersham) and acquired and analyzed using an Odissey Fc Imaging system and the Image StudioÊ software (LiCorBiosciences).

Total cholesterol and triglyceride levels in the plasma: The CH and TG plasma levels were determined using enzymatic kits (Sentinel).

Plasma phospholipids profile: The phospholipid distributions in the FPLC-fractioned plasma were determined using an enzymatic kit (BL chimica).

Cholesterol Efflux Capacity (CEC): The CEC was evaluated with a radioisotopic assay that quantified the total cholesterol efflux mediated by pathways of known relevance in macrophages, i.e., ATP binding cassette transporter A1 (ABCA1) (Khera et al., 2011). J774 cells derived from a murine macrophage cell line were plated and radiolabeled for 24h with 2 εCi/mL of ^{3}H-cholesterol. ABCA1 was up-regulated by an 18-hour incubation with 0.3 mM of 8-(4-chlorophenylthio)-cyclic AMP (cAMP) (Favari et al., 2004b). Subsequently, the efflux medium containing 0.5% whole plasma from either SYN or LERKO female mice in either the P or E phase was added for 4 hours (to prevent serum lipoprotein remodeling at room temperature, all plasma aliquots were defrosted in ice immediately before use). All steps

were performed in the presence of 2 εg/ml of CP113,818 (an acylï coenzyme A:cholesterol acyl-transferase inhibitor) to ensure all cholesterol was in the free form (Zimetti et al., 2006). Liquid scintillation counting was used to quantify the efflux of radioactive cholesterol from the cells. The total radioactive cholesterol incorporated by cells was calculated by isopropanol extraction of control wells that were not exposed to plasma. The CEC is expressed as the percentage of the radioactivity released to the medium over the total radioactivity incorporated by the cells. To minimize the intra-assay variability, every sample was run in triplicate, and the average values and standard deviations were calculated for each percentage of efflux obtained (Favari et al., 2013). cAMP-induced ABCA1 expression was verified by the increase in efflux in response to 10 µg/mL of apoA-I that was used as an ABCA1-specific extracellular acceptor (Favari et al., 2004a). A pool of plasma was used as a reference standard to correct for the inter-assay and intra-assay variabilities (Adorni et al., 2012). The calculated mean intra- and inter-assay coefficients of variation were <6% and <4%, respectively.

Metabolomic analysis: For the LC-MS analysis, 45 εL of plasma was spiked with 5 εL of internal standard (Reserpine) at a known concentration (10 ng/mL) to control the reproducibility of the sample preparation. 100 εL of CH_3CN were added to the spiked plasma to precipitate proteins, and the sample was centrifuged. 120 εL of supernatant was collected and spiked with 100 εL of Bruker Tune mix solution (Bruker Daltonics) to check the LC-MS signal stability during the analysis. The samples were dried with a Thermo Fisher SpeedVac and resuspended in 50 εL of H_2O/CH_3OH (1:1). Before mass spectrometric analysis, analyte separation was achieved with an Ultimate 3000 UPLC (Thermo Fisher) liquid chromatography apparatus using a PFP 50x2.1 1.9µm chromatographic column (Thermo Fisher); the mobile phases were as follows: A) H_2O + 0.2% HCOOH, and B) CH_3OH. A binary gradient was used; 2% of B was maintained for 2 minutes, at 3 minutes, the B% was raised to 30. After an additional 5 minutes, the B% was brought up to 80%, and this % was maintained for 4 minutes; 2% B was reached in 1 minute, and the column was re-equilibrated in the initial conditions for 2 minutes. The chromatographic flow was 0.55 mL/min. The injection volume was 10 µL. Instrument analysis was performed with a QTOF mass analyzer (Bruker Daltonics) coupled to an ESI source and operated in positive ion mode. Full-scan spectra were acquired over the range of 100-700 m/z. The following ion source parameters were used: ESI capillary voltage, 3000 volts; dry gas flow rate, 2 L/min; Dry gas temperature, 300°C; and nebulizer gas flow rate, 80 psi. The data were converted to the mzXML format (Pedrioli et al., 2004) prior to data elaboration analysis using the Bruker Daltonics Data Analysis software. A XCMS algorithm (Smith et al., 2006) was employed in conjunction with an R statistical elaboration package (Bates D, 2014) to obtain both m/z discriminant p values (t test) and to perform the cluster analysis.

Cell cultures and transfections: HeLa cells were co-transfected using Lipofectamine LTX and Plus Reagent (Life Technologies) with 8 ng of the LXRŬ cDNA plasmids (pCMXhLXRalpha) 400 ng of the luciferase reporter (ptk3xLXRE), or ABCA1 luciferase reporter (puc18-mABCA1-pro-luc) or SREBP-1c luciferase reporter (SREBP1C-3000-luc), and 40 ng of б-galactosidase (pCMVbetaGal) in the absence/presence of increasing (ranging from 0.1 to 6.4 ng) concentrations of ERŬ (pCMVhERŬ). HepG2 cells were co-transfected by using Lipofectamine LTX and Plus Reagent with 16 ng of the plasmids coding for PPARŬ (pSG5plHPPARŬWT), 800 ng of the plasmids coding for PPAR luciferase reporter (pMAR(PPRE)5xTkLUC-MAR) and 40 ng of the plasmid pCMVbetaGal in the absence/presence of increasing concentrations of ERŬ (0.8-1.6-3.2-6.4 ng). The cells were then treated with vehicle or with the respective agonists (10 µM T0901317 for LXRŬ and 10 µM WY-14,643 for PPARŬ) in the absence/presence of 10 nM E_2 and of 1 µM ICI 182,780 (Sigma-Aldrich). After 24 hours, the cells were harvested, and luciferase enzymatic activity was measured with a luminometer (Glomax, Promega). The data were normalized to the total protein content (measured by the BCA method to avoid interfering effects) and to б-galactosidase for the transcription efficiency analyses. The data are expressed as RLU/µg proteins.

Co-regulator Recruitment by fluorescence resonance energy transfer (FRET) Assays: The ligand-binding domains (LBDs) of human LXRŬ and ERŬ were expressed as His-tagged proteins using the pET30 and pET15b vectors, respectively. Briefly, freshly transformed *E. coli* BL21 DE3 were grown in 2 L2xTY medium with 30 εg/mL of kanamycin for LXRŬ expression and 100 εg/mL of ampicillin for ERŬ expression at 37°C to an OD of 0.6-0.8. The cultures were then induced with 0.5 mM isopropyl-б-D-thiogalactopyranoside (IPTG) and further incubated at 20°C for 20h. The cells were harvested and resuspended in 40 mL of lysis buffer (50 mM Tris-HCl pH 8, 500 mM NaCl, 1% Triton X-100, 0.1% б-mercaptoethanol, 10% glycerol and protease inhibitors). The cells were sonicated, and the soluble fractions were isolated by centrifugation (35000g at 4°C for 20 min). The supernatants were incubated with Ni^{+2}-nitrilo-triacetic acid slurry for 60 min at 4°C. The slurries were washed, and the protein was recovered in 2 mL of elution buffer (50 mM Tris-HCl pH 8, 150 mM NaCl, 250 mM imidazole, 1 mM TCEP and 10%glycerol). The proteins were then dialyzed over the same buffer without imidazole, quantified and purity on SDS-PAGE.
Fluorescence energy transfer assays were performed to evaluate peptide recruitment in 384-well plates in a final volume of 10 εL. A mix of 8 ng of human LXRŬ or ERŬ LBD, 0.8 ng of Europium-labeled anti-His antibody (Perkin Elmer) and 100 ng allophycocyanin (APC)-labeled streptavidin (Perkin Elmer) in a FRET buffer containing 50 mM Tris pH 7.5, 50 mM KCl, 1 mM DTT and 0.1% free fatty acid BSA was prepared. T0901317 and 17б-estradiol were added to the mix at concentrations that ensured the saturation of the receptors. The biotinylated peptides were added in 12-point

dose response curves starting at 7 εM. The reactions were equilibrated for 1 hour at room temperature and then measured in an Envision multi-plate reader using excitation and emission wavelengths of 340 and 615 nm, respectively. The ratio between 665 nm (APC signal) and 615 nm (Europium signal) was used to evaluate the peptide recruitment on the receptors. The peptide sequences used were the following: biotin-CPSSHSSLTERHKILHRLLQEGSPS-COOH for SRC-1 (spanning aminoacids 676-700, reference number NP_671766), biotin-DGTPPPQEAEEPSLLKKLLLAPANT-COOH for PGC-1α (spanning aminoacids 130-154, reference number NP_037393), biotin-LERNNIKQAANNSLLLHLLKSQTIP-COOH for RIP140 (spanning aminoacids 366-390, reference number NP_003480), biotin-SGNLVPDAASKHKQLSELLRGGSGS-COOH for CBP/P300 (spanning aminoacids 56-80, reference number NP_004371), biotin-GSTHGTSLKEKHKILHRLLQDSSSPVD-COOH for TIF2 (spanning aminoacids 676-702, reference number NP_006531), biotin-PVSSMAGNTKNHPMLMNLLKDNPAQ-COOH for TRAP220 (spanning aminoacids 631-655, reference number NP_038662), and biotin-SFADPASNLGLEDIIRKALMGSFDD-COOH for NcoR (spanning aminoacids2253ï 2277, reference number NP_006302). For competitive assays, the ERŬprotein was cleaved by thrombin to remove the histidine tag and added to a mix containing the previously described amount of LXRŬ to create 12-point dilution curves (starting from 28 ng of ERŬ). T0901317 at 30 nM (the experimentally determined EC50, data not shown) and the indicated coactivator at the EC50 were determined.

ChIP-qPCR: The primer sequences used in ChIP-qPCR analysis are the following:

**Gene**	**Forward**	**Reverse**
Abcg5	5´-CTCTGGACTCAGAAAGGGCA-3´	5´-TCAGTTAAAGCTGCCCTGGA-3´
Abca1	5´-AAACTGAGAGGGCAGGATGA-3´	5´-TGGAAGACTGTGAAGGCTGT-3´
Cyp7a1	5´-CCTTGAACTAAGTCCATCTTCTC-3´	5´-CCAGCTTTGAATGTTATGTCAG-3´
Cyp27a1	5´-AAGATGGATAGGGCAGAAAG-3´	5´-CTGGAACAGGACAGAATCAG-3´
Shp	5´-AGGGGCTCTGAATGATCTTC-3´	5´-GGGGACTTTGCTCACTGTAC-3´
Srebp-1c	5´-GATCAAAGCCAGACGCCGT-3´	5´-CATCCCCGAAAAGAGCCTG-3´
FoxL2	5´-GCTGGCAGAATAGCATCCG-3´	5´-TGATGAAGCACTCGTTGAGGC-3´

SUPPLEMENTAL REFERENCES

Adorni, M.P., Zimetti, F., Puntoni, M., Bigazzi, F., Sbrana, F., Minichilli, F., Bernini, F., Ronda, N., Favari, E., and Sampietro, T. (2012). Cellular cholesterol efflux and cholesterol loading capacity of serum: effects of LDL-apheresis. J Lipid Res *53*, 984-989.

Bates D, C.J., Dalgaard P, Falcon S, Gentleman R, et al. (2014). r-project.

Favari, E., Lee, M., Calabresi, L., Franceschini, G., Zimetti, F., Bernini, F., and Kovanen, P.T. (2004a). Depletion of pre-beta-high density lipoprotein by human chymase impairs ATP-binding cassette transporter A1- but not scavenger receptor class B type I-mediated lipid efflux to high density lipoprotein. J Biol Chem *279*, 9930-9936.

Favari, E., Ronda, N., Adorni, M.P., Zimetti, F., Salvi, P., Manfredini, M., Bernini, F., Borghi, C., and Cicero, A.F. (2013). ABCA1-dependent serum cholesterol efflux capacity inversely correlates with pulse wave velocity in healthy subjects. J Lipid Res *54*, 238-243.

Favari, E., Zanotti, I., Zimetti, F., Ronda, N., Bernini, F., and Rothblat, G.H. (2004b). Probucol inhibits ABCA1-mediated cellular lipid efflux. Arterioscler Thromb Vasc Biol *24*, 2345-2350.

Khera, A.V., Cuchel, M., de la Llera-Moya, M., Rodrigues, A., Burke, M.F., Jafri, K., French, B.C., Phillips, J.A., Mucksavage, M.L., Wilensky, R.L., *et al.* (2011). Cholesterol efflux capacity, high-density lipoprotein function, and atherosclerosis. N Engl J Med *364*, 127-135.

Pedrioli, P.G., Eng, J.K., Hubley, R., Vogelzang, M., Deutsch, E.W., Raught, B., Pratt, B., Nilsson, E., Angeletti, R.H., Apweiler, R., *et al.* (2004). A common open representation of mass spectrometry data and its application to proteomics research. Nat Biotechnol *22*, 1459-1466.

Rozowsky, J., Euskirchen, G., Auerbach, R.K., Zhang, Z.D., Gibson, T., Bjornson, R., Carriero, N., Snyder, M., and Gerstein, M.B. (2009). PeakSeq enables systematic scoring of ChIP-seq experiments relative to controls. Nat Biotechnol *27*, 66-75.

Smith, C.A., Want, E.J., O'Maille, G., Abagyan, R., and Siuzdak, G. (2006). XCMS: processing mass spectrometry data for metabolite profiling using nonlinear peak alignment, matching, and identification. Anal Chem *78*, 779-787.

Villa, A., Della Torre, S., Stell, A., Cook, J., Brown, M., and Maggi, A. (2012). Tetradian oscillation of estrogen receptor alpha is necessary to prevent liver lipid deposition. Proceedings of the National Academy of Sciences of the United States of America *109*, 11806-11811.

Zimetti, F., Weibel, G.K., Duong, M., and Rothblat, G.H. (2006). Measurement of cholesterol bidirectional flux between cells and lipoproteins. J Lipid Res *47*, 605-613.
