# Supplementary material for: An Essential Role for Liver ERα in Coupling Hepatic Metabolism to the Reproductive Cycle
Source: Cell Rep. 2016 Mar 31;15(2):360–71. doi: 10.1016/j.celrep.2016.03.019 (PMC4835581; doi:10.1016/j.celrep.2016.03.019)
Supplement: Document S2. Article plus Supplemental Information [file mmc2.pdf]

Cell Reports

Article

An Essential Role for Liver ERα in Coupling Hepatic Metabolism to the Reproductive Cycle

Graphical Abstract

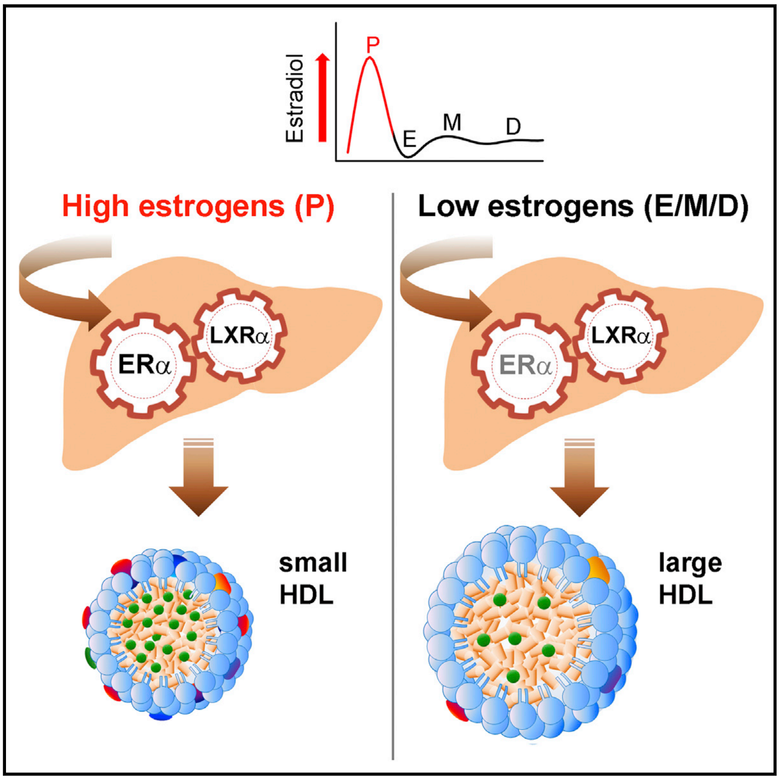

Highlights

- Liver ERα couples lipid metabolism to the reproductive cycle
- Liver ERα regulates lipoprotein remodeling enzymes
- Liver ERα elicits the cyclic synthesis of highly efficient HDL
- Liver ERα regulates female hepatic metabolism through interaction with LXRα

Authors

Sara Della Torre, Nico Mitro, Roberta Fontana, ..., Nina Henriette Uhlenhaut, Laura Calabresi, Adriana Maggi

Correspondence

adriana.maggi@unimi.it

In Brief

Della Torre et al. show that ERα activity in the mouse female liver is essential for balanced lipid and cholesterol metabolism. Lack of liver ERα activation in the case of ovarian failure leads to metabolic dysfunction that may be linked to post-menopausal disease.

Della Torre et al., 2016, Cell Reports *15*, 360–371
April 12, 2016 ©2016 The Authors
http://dx.doi.org/10.1016/j.celrep.2016.03.019

CellPress

OPEN ACCESS
CellPress

An Essential Role for Liver ERα in Coupling Hepatic Metabolism to the Reproductive Cycle

Sara Della Torre,[1,2] Nico Mitro,[2] Roberta Fontana,[2,3] Monica Gomaraschi,[2] Elda Favari,[4] Camilla Recordati,[5] Federica Lolli,[1,2] Fabiana Quagliarini,[6] Clara Meda,[1,2] Claes Ohlsson,[7] Maurizio Crestani,[2] Nina Henriette Uhlenhaut,[6] Laura Calabresi,[2] and Adriana Maggi[1,2,*]
[1]Center of Excellence on Neurodegenerative Diseases, University of Milan, 20133 Milan, Italy
[2]Department of Pharmacological and Biomolecular Sciences, University of Milan, 20133 Milan, Italy
[3]Department of Drug Discovery and Development, Italian Institute of Technology, 16163 Genova, Italy
[4]Department of Pharmacy, University of Parma, 43121 Parma, Italy
[5]Mouse and Animal Pathology Laboratory, Fondazione Filarete, 20139 Milan, Italy
[6]Helmholtz Diabetes Center (HDC) and German Center for Diabetes Research (DZD), Helmholtz Zentrum Muenchen, 85764 Munich-Neuherberg, Germany
[7]Centre for Bone and Arthritis Research and Department of Internal Medicine and Clinical Nutrition, Institute of Medicine, Sahlgrenska Academy, University of Gothenburg, 40530 Gothenburg, Sweden
*Correspondence: adriana.maggi@unimi.it
http://dx.doi.org/10.1016/j.celrep.2016.03.019

SUMMARY

Lipoprotein synthesis is controlled by estrogens, but the exact mechanisms underpinning this regulation and the role of the hepatic estrogen receptor α (ERα) in cholesterol physiology are unclear. Utilizing a mouse model involving selective ablation of ERα in the liver, we demonstrate that hepatic ERα couples lipid metabolism to the reproductive cycle. We show that this receptor regulates the synthesis of cholesterol transport proteins, enzymes for lipoprotein remodeling, and receptors for cholesterol uptake. Additionally, ERα is indispensable during proestrus for the generation of high-density lipoproteins efficient in eliciting cholesterol efflux from macrophages. We propose that a specific interaction with liver X receptor α (LXRα) mediates the broad effects of ERα on the hepatic lipid metabolism.

INTRODUCTION

The liver plays a unique, central role in the regulation of fatty acid (FA) and cholesterol (CH) metabolism. Alterations in the homeostatic control of lipid metabolism have severe pathological repercussions, as the accumulation of fat in the hepatocytes is associated with non-alcoholic fatty liver disease (NAFLD), metabolic disease, and cardiovascular disease (CVD).

An emerging theme in the regulation of hepatic lipid metabolism is the involvement of estrogens and associated receptors. Prior work has demonstrated that the liver is a major target for estrogens, and the transcriptional activity of hepatic estrogen receptors (ERs) is strictly associated with the reproductive cycle (Ciana et al., 2003) and nutritional status (Ciana et al., 2005; Della Torre et al., 2011). Several lines of evidence indicate that estrogens are involved in the prevention of hepatic fat deposits: (1) estrogens reduce hepatic lipid synthesis and increase the transport of triglycerides (TGs); (2) sex and the reproductive state influence the prevalence of NAFLD and the degree of fibrosis in patients with its more severe form, non-alcoholic steatohepatitis (NASH); and (3) pathologies characterized by ovarian dysfunction, such as polycystic ovary syndrome and Turner syndrome, are generally associated with NAFLD (Clegg, 2012; Gambarin-Gelwan et al., 2007; Gutierrez-Grobe et al., 2010; Ostberg et al., 2005).

Although a number of studies in experimental animals and in women have addressed the beneficial role of estrogen signaling in counteracting fatty liver disease/NAFLD and CVD (Barsalani et al., 2010; Della Torre et al., 2014; Roeters van Lennep et al., 2002), the exact mechanisms underpinning the increased incidence of NAFLD following menopause and ovariectomy (OVX) and their relation with the etiology of CVD remain unclear. Understanding the physiology of estrogen-dependent regulation of energy metabolism in the female liver is necessary for the development of new therapies, particularly for the treatment of metabolic disorders associated with menopause and ovarian dysfunction.

The model systems applied so far, i.e., OVX and total body ER knockout (KO), have not been able to distinguish between systemic and intra-hepatic estrogen effects. To overcome this issue, we generated a conditional liver KO of ER α (ERα) (Esr1), the predominant ER isoform in liver (LERKO). LERKO mice maintain a regular reproductive cycle (Della Torre et al., 2011) and, therefore, provide a unique opportunity to study the consequences of the lack of liver ERα in the context of female reproductive physiology.

The aim of the present study was to investigate the physiological role of hepatic ERα in the control of lipid metabolism in females and to examine the extent to which liver ERα can be considered a target for hepatic metabolic dysfunction therapy.

This is an open access article under the CC BY license (http://creativecommons.org/licenses/by/4.0/).

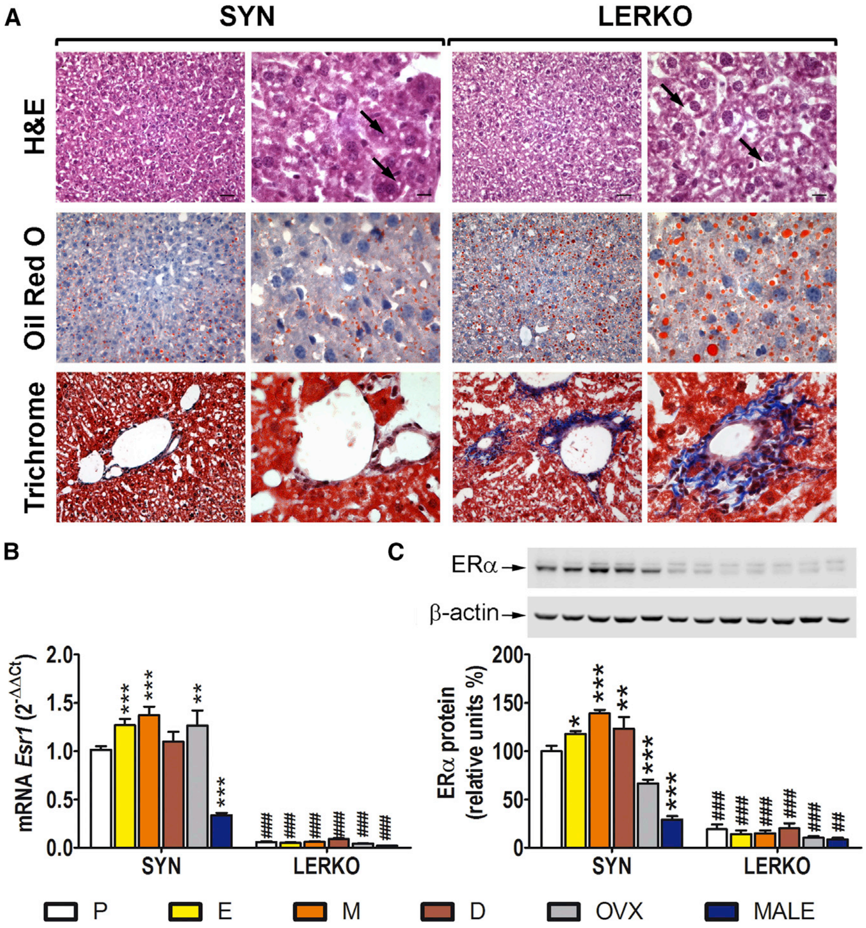

Figure 1. Liver Histology and Measurement of *Esr1* Expression in the SYN and LERKO Mice

(A) Liver histology. Top: H&E staining; the black arrows highlight hepatocellular vacuolar degeneration. Center: oil red O staining plus H&E (neutral fats are stained orange red, and the nuclei are shown in blue). Bottom: Masson's trichrome staining with aberrant collagen deposits (blue); the hepatocyte cytoplasm is red, and the nuclei are dark red-black structures within cells. For both SYN and LERKO: scale bar for left columns, 33 μm; scale bar for right columns, 10.6 μm.

(B) Quantitative analysis of *Esr1* mRNA in the livers of 3-month-old cycling females measured by real-time PCR; OVX for 30 days and age-matched males. The data indicate mean ± SEM; n = 6 ÷ 12; the experiment was repeated three times.

(C) Representative western blot and semi-quantitative analysis of ERα protein in liver extracts. The data indicate mean ± SEM; n = 5. The experiment was repeated three times.

*$p < 0.05$, **$p < 0.01$, and ***$p < 0.001$ versus SYN at P; ##$p < 0.01$ and ###$p < 0.001$ versus SYN.

RESULTS

The Selective Ablation of Esr1 Leads to an Aberrant Deposition of Fat and Collagen in the Female Mouse Liver

The effects of liver *Esr1* ablation were initially studied in fertile females euthanized at 10 months of age. The livers of syngenic (SYN) and LERKO mice were dissected for morphological examination based on a combination of staining procedures (Figure 1A; Table S1). H&E staining revealed a variable degree (from mild to marked) of hepatocellular vacuolar degeneration. Although overt effects of *Esr1* ablation were not immediately evident, in the LERKO mice, the vacuolization was slightly more marked, which was suggestive of changes in fat deposits. Such changes were further suggested by the observation that, overall, the oil-red-O-stained lipid droplets were larger in the LERKO than in the SYN mice and that Masson's trichrome staining of the LERKO livers revealed portal infiltration of mononuclear leukocytes and portal or centrilobular collagen deposition. Quantitative analyses demonstrated that the livers of the LERKO mice exhibited increased oil red O staining (+112%; Figure S1; Table S1) and a greater expression of genes involved in the inflammatory process and collagen deposition (Figure S2).

Interestingly, when the study was carried out in 10-month-old SYN males, we observed that the lipid accumulation was 4.4-fold higher than in females of the same age; however, the effect of *Esr1* ablation in males (+28%) was less pronounced than in females (+112%; Figure S1), thus suggesting a sexually dimorphic role of hepatic ERα in the control of liver lipid homeostasis. Further investigations revealed that, in males, the content of ERα protein (Figure 1C) was, indeed, significantly lower than in females. However, the most remarkable observation was that the concentrations of ERα mRNA and protein in females changed significantly across the different phases of the estrous cycle; ERα mRNA and protein content was the lowest at proestrus (P) (−26% and −39%, respectively, versus metestrus [M]) and OVX further decreased ERα protein (−52% versus M).

These results might explain the minor effect of liver ERα ablation in males and led us to further investigate the role of liver ERα in lipid metabolism in females in relation to the different phases of the reproductive cycle.

Lipid and Lipoprotein Metabolism in the Liver: Effects of the Reproductive Cycle and Esr1 Ablation

The following series of findings in female mouse liver indicated a tight coupling between the reproductive cycle and CH metabolism (Figure 2): total CH content oscillated with the estrous cycle, and free/total CH was the lowest during diestrus (D); in this phase of the cycle, the ratio between cholesteryl esters (CEs) and total CH was the highest. CH catabolism was also regulated as indicated by measurements of bile acid (BA) contents in the feces, which were lower at estrus (E) and M than at P and D. These changes were not observed in the LERKO mice, which

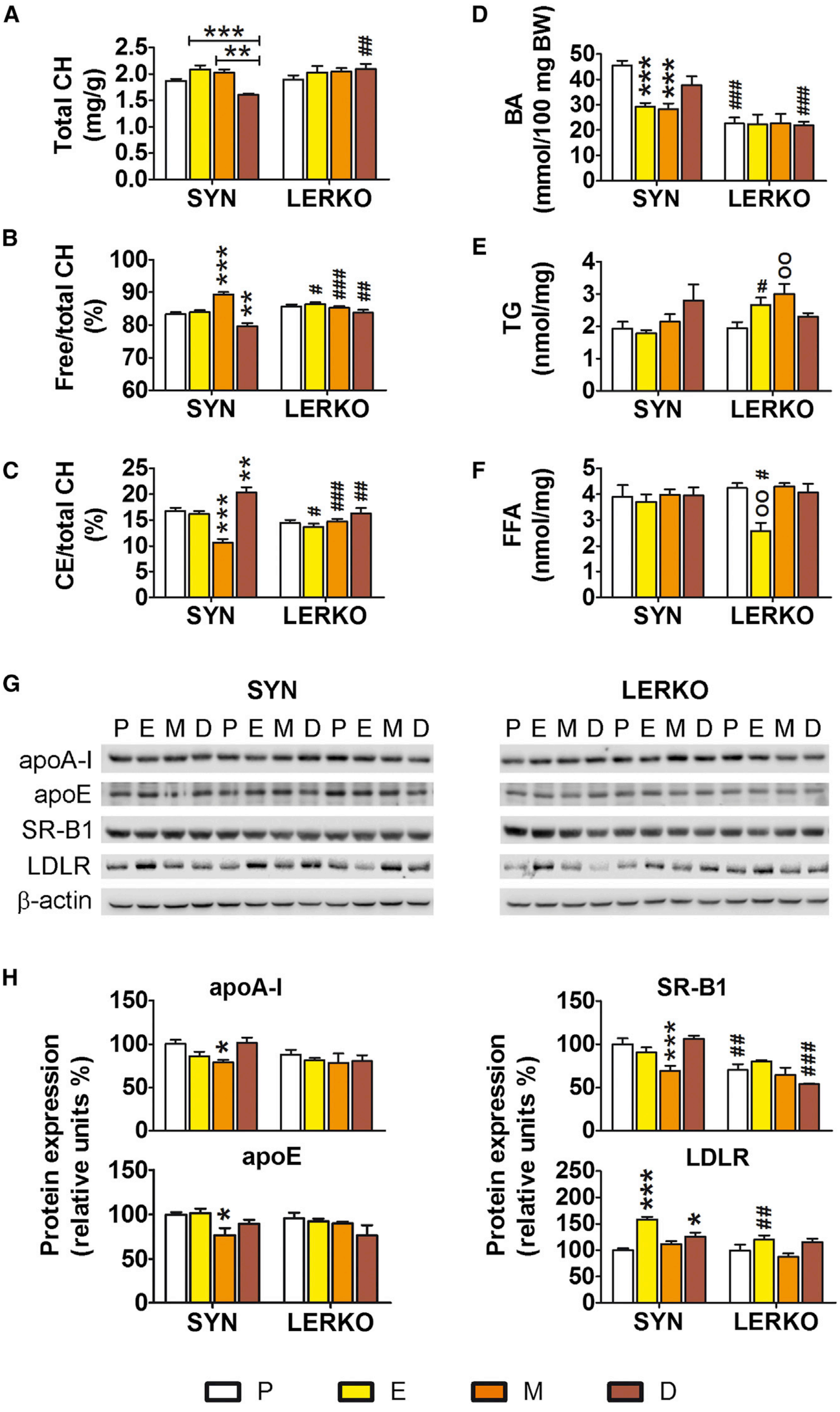

Figure 2. Effect of Estrous Cycle Progression on Liver CH Metabolism in the SYN and LERKO Female Mice

Liver extracts were obtained from the livers of 3-month-old female mice.

(A–C) Total CH content (A). Free CH (B) and CE (C) content expressed as a percentage of the total CH. The data indicate mean ± SEM; n = 10.

(D) BA content measured in the feces. The data indicate mean ± SEM; n = 10.

(E and F) TG (E) and FFA (F) liver contents. The data indicate mean ± SEM; n = 5.

(G) Representative western blotting analyses of the contents of apo-AI, apo-E, SR-B1, and LDLR in liver extracts.

(H) Semiquantitative analyses of blotting with antibodies anti-apo-AI, -apo-E, -SR-B1, and -LDLR. The data indicate mean ± SEM of six animals. The experiment was repeated twice.

$^{*}p < 0.05$, $^{**}p < 0.01$, and $^{***}p < 0.001$ versus SYN at P; $^{OO}p < 0.01$ versus LERKO at P; $^{\#}p < 0.05$, $^{\#\#}p < 0.01$, and $^{\#\#\#}p < 0.001$ versus SYN.

led to the conclusion that, in the absence of liver ERα, hepatic CH metabolism and ovarian activity are uncoupled (Figures 2A–2D).

In contrast, measurements of hepatic TG content and free fatty acids (FFAs) revealed no influence of the estrous cycle in the SYN mice, whereas in the LERKO livers, TGs accumulated during M and FFA decreased during E, which highlighted a role of ERα in the maintenance of TGs and FFA homeostasis in the liver (Figures 2E and 2F).

Next, we studied the proteins involved in CH transport: in SYN mice, both synthesis and uptake of high-density lipoproteins (HDLs) appeared to be the lowest at M, as indicated by the content of apolipoprotein-AI (apo-AI) and apolipoprotein-E (apo-E) and their receptor (scavenger receptor class B member; SR-B1). A different pattern was observed for the low-density lipoprotein (LDL) receptor (LDLR), the hepatic content of which was the highest at E. Again, none of these cycle-related changes were observed in the LERKO mice (Figures 2G and 2H).

To verify whether the differences observed in the estrous cycle of SYN and LERKO females were not caused by increased levels or altered fluctuation of 17β-estradiol (E_2) in the plasma of LERKO, we measured the circulating levels of the hormone in each phase of the cycle by gas chromatography-tandem mass spectrometry (GC-MS/MS), and we analyzed uterus weight as a well-known quantitative bioassay for circulating estrogens. Figure S3A shows that the circulating levels of E_2 fluctuate similarly in SYN and LERKO; the same is true for the weight of the uterus (Figure S3B). This was expected, as we did not observe any fertility phenotype in LERKO and demonstrated that the absence of liver ERα was the major cause of the changes prior described.

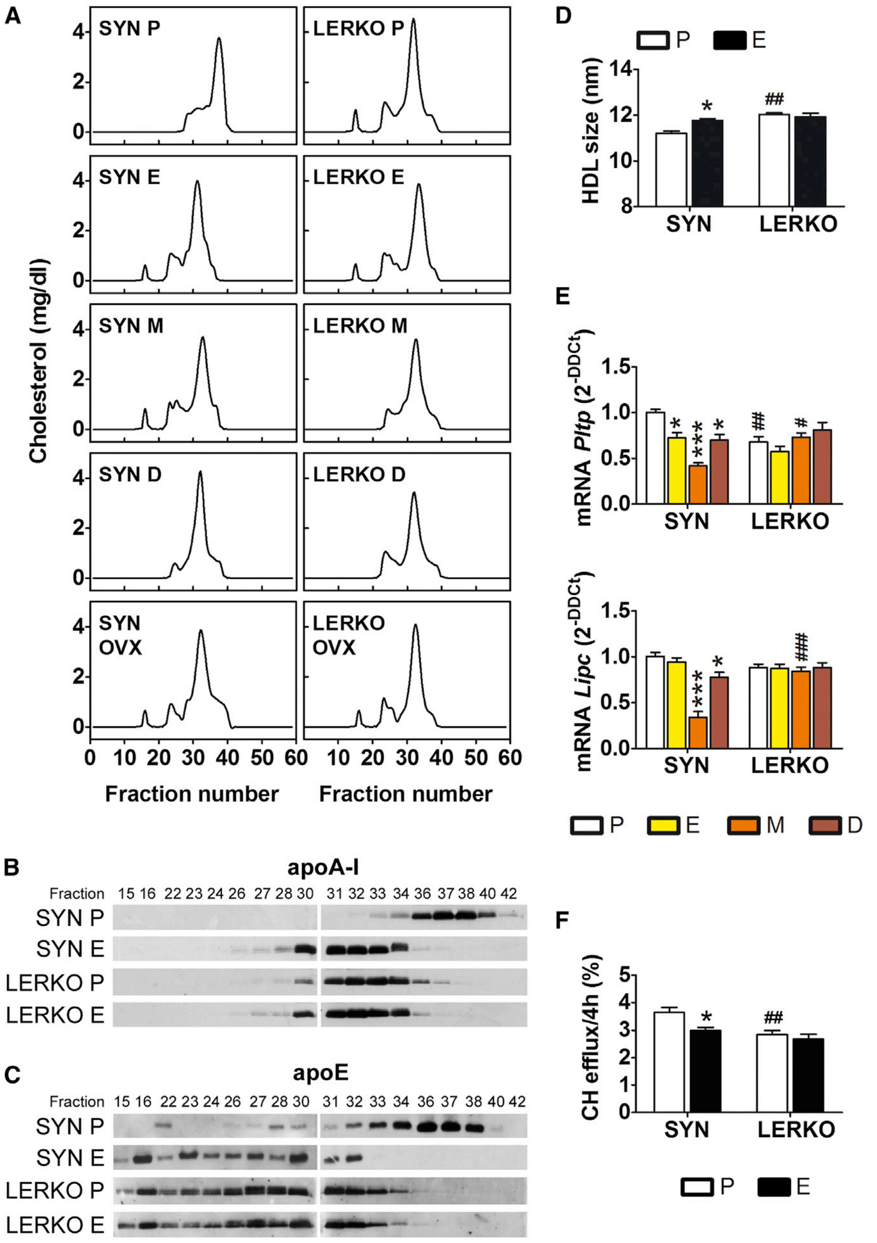

Figure 3. CH Profiles and Lipoprotein Analyses of the Plasma of the SYN and LERKO Females

Plasma was obtained from SYN and LERKO females at 3 months of age euthanized at different phases of the estrous cycle or 30 days after OVX.

(A) Representative profile of the total CH content (expressed as milligrams per deciliters) in the fractions of plasma separated by FPLC. The experiment was repeated three times with six different animals in each experimental group.

(B and C) Western blot for apo-AI (B) and apo-E (C) in the FPLC fractions of the plasma at P and E.

(D) Sizes of HDLs (d = 1.063–1.21 g/ml) purified by sequential ultracentrifugation from pooled plasma samples. The data indicate mean ± SEM; n = 3 pools of plasma (each pool was composed of the plasma of six mice).

(E) Real-time PCR quantitative analyses of the liver mRNA contents of *Pltp* (top) and *Lipc* (bottom). The data indicate mean ± SEM; n = 6. The experiment was repeated twice.

(F) CEC as measured by radioisotopic assay in J774 cells pre-radiolabeled with ^{3}H-CH and incubated with plasma from either SYN or LERKO females at P or E. The data are expressed as the percentage of the radioactivity released into the medium over the total radioactivity incorporated by the cells. The data indicate mean ± SEM; n = 10.

*p < 0.05 and ***p < 0.001 versus SYN at P; $^{\#}$p < 0.05, $^{\#\#}$p < 0.01, and $^{\#\#\#}$p < 0.001 versus SYN.

Liver ERα and HDL Remodeling and Function

When we analyzed total plasma CH (Figure S4), we did not observe changes associated with the estrous cycle in either SYN or LERKO mice. Conversely, the plasma TG levels appeared to be regulated by cycle-dependent factors, because oscillations in their levels were observed in both SYN and LERKO mice. The TG content was slightly decreased in the SYN mice at M (−17%) and increased in the LERKO mice at D (+20%).

CH distribution among plasma lipoproteins was analyzed by fast protein liquid chromatography (FPLC). Figure 3A shows that, in the SYN mice, the CH-lipoprotein profile was very reproducible across all phases of the estrous cycle, with the exception of P, in which we observed a significant delay in the elution of the HDL peak. Indeed, in the plasma of the SYN mice at E, M, and D, CH eluted in fractions 31–33, whereas at P, the CH eluted in fractions 36–38. This observation suggested that the HDLs were smaller during P. This phenomenon was not observed in the plasma of the LERKO mutants, in which the FPLC profiles of the HDLs were the same in all the phases of the cycle and were superimposable to those of the SYN mice at E, M, and D. These findings provided evidence for the involvement of liver ERα in the generation of a distinct class of HDLs during P (when the circulating estrogens were the highest) and led us to investigate the consequences of OVX. In OVX mice, CH was present in the HDLs (eluting in fractions 31–33) and in the other classes of lipoproteins (very low-density lipoprotein [VLDL] and LDL), showing a CH profile similar to that of E (the phase of the estrous cycle with the lowest concentration of estrogens).

A further characterization of the proteins that eluted with CH proved that the apo-AI protein content was markedly increased in fractions 32 and 33 in all samples, with the exception of those from the SYN mice at P, in which apo-AI was most abundant in fractions 36–38. This finding confirmed that the late-eluting fractions contained small HDL particles. Similar results were obtained when we studied the apo-E distribution; apo-E was found

Table 1. HDL Composition

HDL	Mean ± SEM for Composition (%) SYN P	SYN E	LERKO P	LERKO E
Proteins	50.1 ± 1.0	54.8 ± 0.8*	52.0 ± 0.8	51.7 ± 2.1
PLs	18.4 ± 0.9	19.4 ± 0.7	21.1 ± 2.2	21.1 ± 2.2
Unesterified CH	2.8 ± 0.1	2.1 ± 0.5	3.1 ± 0.6	3.0 ± 0.6
Esterified CH	23.7 ± 0.9	21.2 ± 1.4	21.1 ± 3.9	21.3 ± 1.0
Triglycerides	5.2 ± 0.9	2.5 ± 0.4*	3.0 ± 0.5	3.1 ± 0.4

HDLs (d = 1.063–1.21 g/ml) were purified by sequential ultracentrifugation from the plasma pooled from six mice. Protein, PL, total and unesterified CH (TC and UC), and TG contents were measured as explained in the Experimental Procedures. The data are mean ± SEM; n = 3 pools of plasma. *p < 0.05 versus SYN at P by two-way ANOVA followed by Bonferroni post hoc tests.

in fractions 32–34 in all the plasma samples examined, with the exception of those from P of the SYN mice, in which apo-E appeared in fractions 34–38.

To better characterize the HDLs circulating at P in the SYN mice (P-HDL), we studied their sizes with non-denaturing polyacrylamide gradient gel electrophoresis (GGE) and studied their mass composition. SYN P-HDLs were significantly smaller than those circulating at E and those in the LERKO mice (Figure 3D), and they were characterized by a high TG content (+108% versus E) and a small, but significant, reduced content of proteins (−8.6% versus E). No changes were observed in the amounts of CH (esterified or unesterified) or phospholipids (PLs; Table 1). In the LERKO mice, the compositions of plasma HDLs at P and E were extremely similar and not significantly different from those of the SYN mice at E (Table 1). The analysis of the PL contents of the FPLC eluate gave results consistent with the findings shown in Figure 3A; indeed, a major PL peak was observed around fraction 38 in the SYN mice at P and around fraction 32 in all the other samples (Figure S5). Because circulating lipoproteins undergo significant changes in size due to the activities of specific remodeling enzymes, we studied the effects of the cycle and liver *Esr1* ablation on the expression of the genes that encode PL transfer protein (*Pltp*) and hepatic lipase (*Lipc*). In the SYN mice, the liver content of both enzymes changed with the estrous cycle (the lowest concentrations were observed at M). Again, no change was observed in the LERKO mice (Figure 3E).

These results provided strong evidence that the P-HDLs were structurally dissimilar from the lipoproteins generated during the other phases of the estrous cycle and that the activity of liver ERα was essential for their production. Next, we considered whether the peculiarities of the P-HDL structure reflected key aspects of their function by comparing the capacities of HDLs isolated from the SYN and LERKO mice to elicit CH efflux from macrophages. The HDL CH efflux capacity (CEC) estimates the efficiency of the entire reverse CH transport (RCT) process and is an accepted index of HDL functionality (Rohatgi et al., 2014). We used the murine macrophage cell line J774, which is known to express the ATP binding cassette transporter A1 (ABCA1) upon induction with a cyclic AMP (cAMP) analog (Favari et al., 2013). Figure 3F shows that, in SYN mice, P-HDL efficiency in inducing CH efflux was significantly greater than that of HDL isolated at E (+23%). This cycle-dependent effect was not observed in the LERKO mice; indeed, the functionality of the LERKO HDL was indistinguishable from that of SYN HDL at E.

Overall, these data demonstrated that, during the course of the reproductive cycle, the ability of HDL to elicit RCT changed in relation to plasma estrogen content and hepatic ERα activity. These findings led us to further evaluate the extent to which liver ERα was able to translate ovarian output into changes in liver metabolism via a metabolomics analysis of plasma samples collected at P and E. Figure S6A indicates that the transition from P to E in the SYN mice was associated with changes in 5.3% of all plasma metabolites. In LERKO mice, 34% of these metabolites were not affected by the estrous cycle, which underscored the role of liver ERα in the control of the hepatic endocrine functions. Comparison of the SYN and LERKO samples indicated that the plasma metabolites differed between these groups by 4.9% during P and 2.7% during E, which suggests that 45% of these differences were induced by ligand-dependent activation of liver ERα (see Figure S6B).

Therefore, in addition to lipoproteins, several plasma components were regulated by ERα, further suggesting the involvement of this receptor in the functional coupling of liver to the ovarian cycle. The breadth of ERα effects on lipid metabolism led us to further investigate on the underpinning mechanisms.

Hepatic ERα Cross-Couples with LXRα but Not PPARα

Previously, two ligand-dependent transcription factors, peroxisome proliferator-activated receptor alpha (PPARα) and liver X receptor alpha (LXRα), have been found to act in the liver as the major nutritional sensors and key transcriptional modulators of lipid and carbohydrate metabolism (Bocher et al., 2002; Li and Glass, 2004; Zhang et al., 2012). This led us to ask whether ERα had any effect on the synthesis or activity of these two receptors. No changes in PPARα mRNA or protein contents were observed in the different phases of the reproductive cycle (Figure 4A). Similarly, no changes in the activity of PPARα were attributable to the progression of the estrous cycle, because the liver contents of PPARα target genes, such as carnitine palmitoyltransferase 1A (*Cpt1α*), hydroxyacyl-coenzyme A (CoA) dehydrogenase α (*Hadhα*), acyl-CoA oxidase 1 (*Acox*), and acyl-CoA synthetase long-chain family member 1 (*Acsl1*), were the same in all phases of the cycle (Figure 4B). These results were replicated in the LERKO mice in which the expressions of PPARα and its target genes were superimposable with the patterns observed in the SYN mice. This suggested that the aforementioned effects reported of hepatic ERα on liver metabolism did not occur via PPARα.

In contrast, LXRα expression was modulated by the estrous cycle in the SYN mice. A significant increase of both LXRα mRNA and protein was observed at E (+13% and +45%, respectively, versus P; Figure 4C). The estrous cycle affected also LXRα transcriptional activity, as indicated by the changes in the liver content of the mRNA encoded by its target genes in SYN mice; for most of the LXRα target genes investigated (four of six), the respective mRNA was decreased at M (i.e., ATP-binding cassette sub-family G member 5, *Abcg5*; ATP-binding cassette transporter A1, *Abca1*; CH 7-alpha-hydroxylase, *Cyp7a1*; and sterol 27-hydroxylase, *Cyp27a1*). No cycle-related changes were observed in the expression of small heterodimer partner (*Shp*) and sterol regulatory element binding protein-1c (*Srebp-1c*),

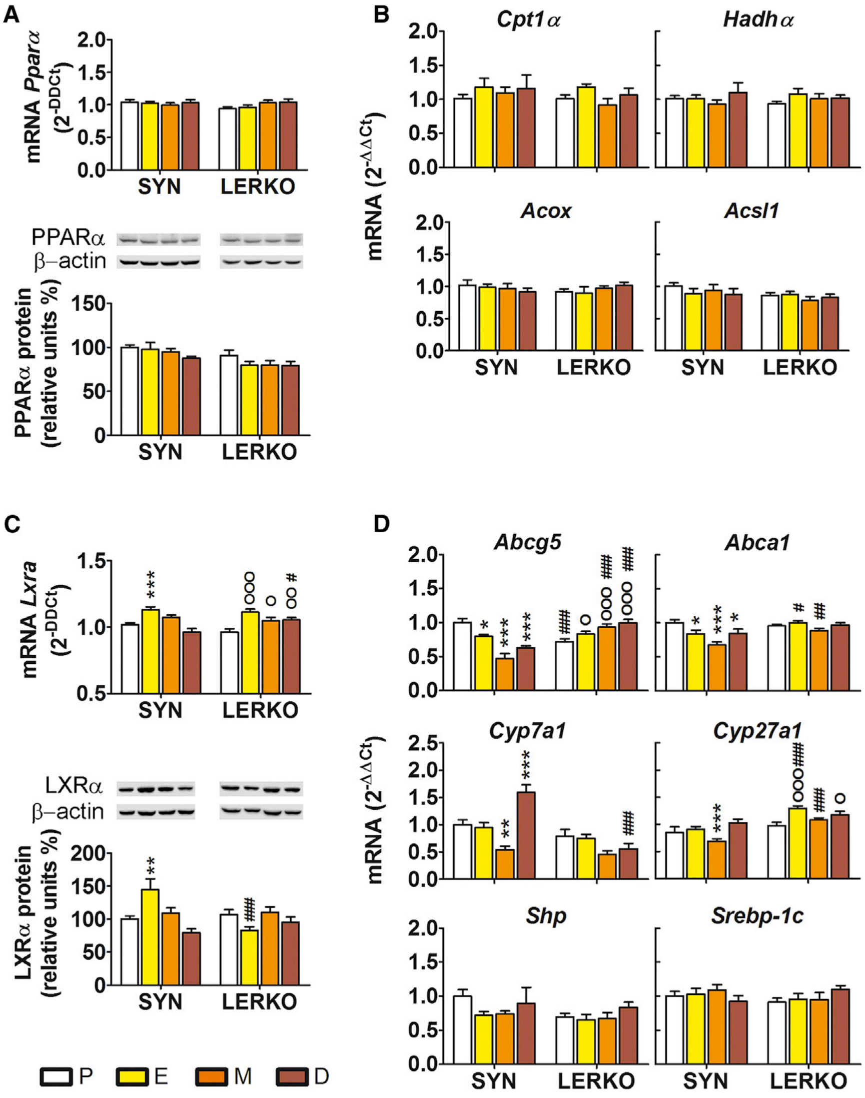

Figure 4. Effect of the Estrous Cycle on PPARα and LXRα Syntheses and Transcriptional Activities

(A) The PPARα mRNA (top) and protein (bottom) liver contents were measured by real-time PCR and western blot analysis.

(B) Real-time PCR quantitative analyses of the PPARα target genes *Cpt1α*, *Hahdα*, *Acox*, and *Acsl1*.

(C) mRNA (top) and protein (bottom) of LXRα contents in liver homogenates from the SYN and LERKO mice.

(D) The mRNA contents of the LXRα target genes *Abcg5*, *Abca1*, *Cyp7a1*, *Cyp27a1*, *Shp*, and *Srebp-1c*.

For all of the real-time PCR analyses, the data indicate mean ± SEM, n = 6. The experiments were repeated three times. *$p < 0.05$, **$p < 0.01$, and ***$p < 0.001$ versus SYN at P; $^{O}p < 0.05$, $^{OO}p < 0.01$, and $^{OOO}p < 0.001$ versus LERKO at P; $^{\#}p < 0.05$, $^{\#\#}p < 0.01$, and $^{\#\#\#}p < 0.001$ versus SYN.

the LXRα target gene master regulator of de novo lipogenesis. The results obtained in the LERKO mice underscored the necessity of ERα for the cycle-dependent expression of these factors, because the liver content of LXRα protein or *Abca1*, *Cyp7a1*, or *Shp* mRNAs was not affected by the progression of the estrous cycle. Curiously, *Cyp27a1* and *Abcg5* exhibited oscillations with the cycle in LERKO too, but the pattern of these fluctuations differed from those of the SYN mice, pointing to the involvement of cycle-dependent factors, which became predominant only in the absence of the hepatic ERα (Figure 4D).

Overall, these data suggested the existence of a physiological, functional cross-coupling between ERα and LXRα in the liver for the regulation of CH metabolism.

Hepatic ERα Interferes with the Transcriptional Activity of LXRα but Not PPARα

The existence of a functional interaction between ERα and LXRα was further investigated in co-transfection studies. Figure 5A shows that the LXRα-dependent activity of the LXRE-Luc promoter was augmented 13.6-fold in the presence of the LXRα-specific agonist T0901317 (T09). Co-transfection with increasing concentrations of ERα in the presence of 10 nM E_2 substantially diminished LXRα transcriptional efficiency (from 9.3- to 5.0-fold at the highest ERα concentration). However, when the ERα antagonist ICI 182,780 was added, ERα inhibition was maintained. This highlighted the possibility of a ligand-independent effect of ERα, which was further demonstrated in transfection experiments performed in the absence of E_2 (data not shown). Remarkably, this effect was specific to LXRα, because the transcriptional activity of PPARα that was stimulated by its agonist WY-14,643 was not altered by the presence of ERα with E_2 or E_2 plus ICI 182,780 (Figure 5B). Next, we asked whether the unliganded ERα was able to interfere with LXRα activity on the ABCA1 and SREBP-1c promoters. Figures 5C and 5D show that ERα interfered with LXRα transcriptional activity on the ABCA1, but not of the SREBP-1c promoter. This was consistent with prior observation (Figure 4D) that the transcription of SREBP-1c in liver was not modulated by the estrous cycle or influenced by the absence of liver ERα.

ERα and LXRα Functional Interaction in Liver

Next, we investigated the hypothesis that ERα-dependent modulation of LXRα transcriptional activity was due to a competition between ERα and LXRα for common co-regulators. The mutual interference of ERα and LXRα in the recruitment of common co-activators was studied using fluorescence resonance energy transfer (FRET; Figures 5E and 5F). In this assay, we observed that the addition of increasing amounts of ERα protein

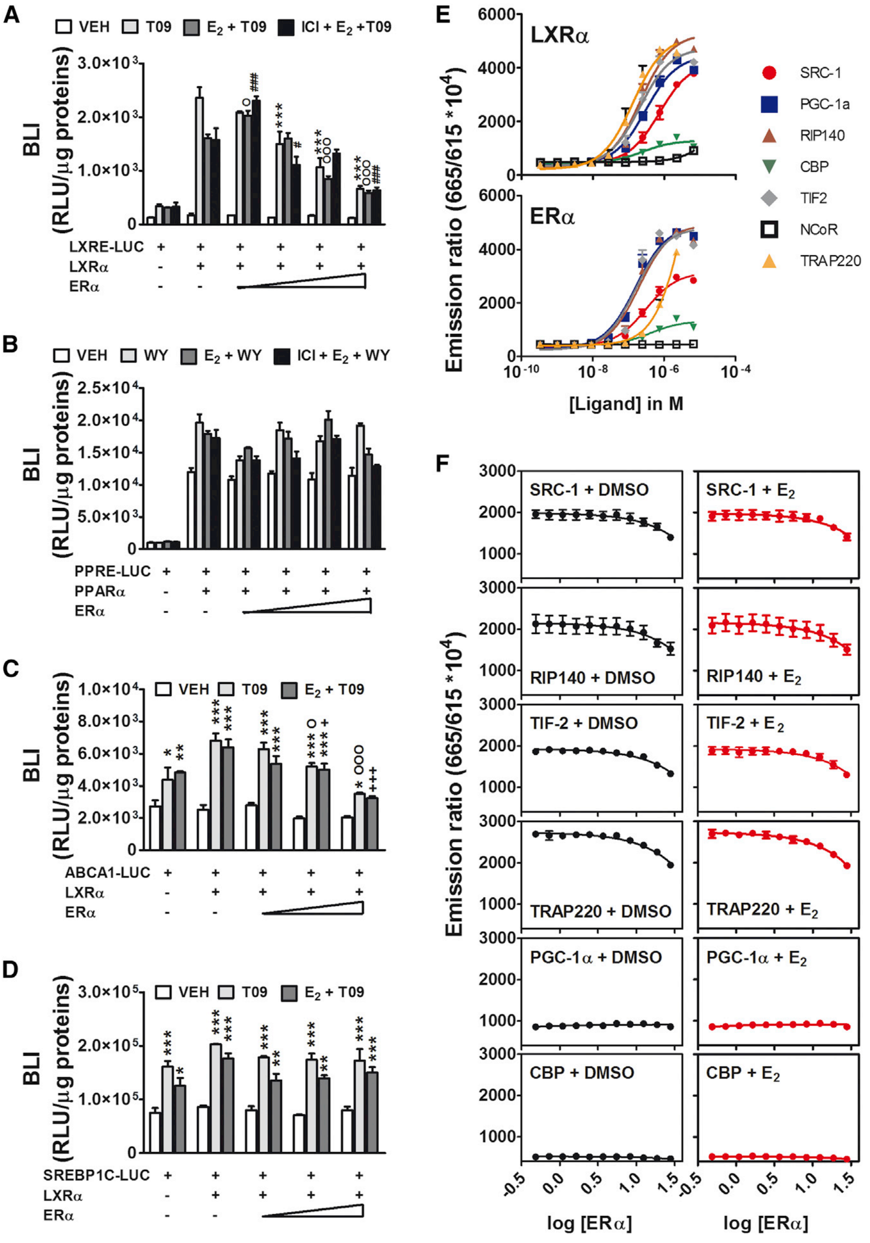

Figure 5. Inhibition of the Transcriptional Activity of LXRα by ERα: In Vitro Studies

(A) HeLa cells were co-transfected with LXRα and the reporter LXRE-Luc in the presence or absence of ERα. Where indicated, LXRα agonist T0901317 (T09), E_2 plus T09, and ERα antagonist ICI 182,780 (ICI) were added. The data indicate mean ± SEM, n = 4; each experiment was repeated three times. VEH, vehicle. ***p < 0.001 versus LXRα/LXRE-Luc+T09; ^{O}p < 0.05 and ^{OOO}p < 0.001 versus LXRα/LXRE-Luc+E_2+T09; $^{\#}$p < 0.05 and $^{\#\#\#}$p < 0.001 versus LXRα/LXRE-Luc+ICI+E_2+T09.

(B) Effect of ERα on the transcriptional activity of PPARα. The cells were co-transfected with PPARα and the reporter PPRE-Luc in the presence or absence of ERα. Where indicated, the cells were treated with the PPARα agonist WY-14,643 (WY), E_2 + WY, and ICI + E_2 + WY. The data indicate mean ± SEM, n = 4; the experiment was repeated three times.

(C and D) HeLa cells were co-transfected with LXRα and the reporter ABCA1-Luc (C) or SREBP1C-Luc (D) in the presence or absence of ERα. Treatments were done with vehicle, T09, or E_2 + T09. The data indicate mean ± SEM, n = 4; each experiment was repeated three times. *p < 0.05, **p < 0.01, and ***p < 0.001 versus VEH; ^{O}p < 0.05 and ^{OOO}p < 0.001 versus LXRα/ABCA1-Luc+T09; $^{+}$p < 0.05 and $^{+++}$p < 0.001 versus LXRα/ABCA1-Luc+E_2+T09.

(E) Identification of the co-activators of the LXRα (top) and ERα (bottom) proteins by FRET. SRC-1, PGC-1α, RIP140, CBP, TIF2, nuclear receptor corepressor (NCoR), and TRAP220. The data indicate mean ± SEM, n = 2; the experiment was repeated twice.

(F) FRET analysis of the changes in the recruitment of co-activators by LXRα in the presence of increasing amounts of ERα stimulated with DMSO (dark lanes) or 5 nM E_2 (red lanes). The data indicate mean ± SEM, n = 2; the experiment was repeated three times.

BLI, bioluminescence imaging; RLU, relative light units.

progressively augmented the competition with LXRα for steroid receptor coactivator 1 (SRC-1), nuclear receptor interacting protein (RIP140), transcriptional intermediary factor 2 (TIF2), and thyroid hormone receptor-associated protein (TRAP220). No effect was seen in the recruitment of PPARγ coactivator 1-alpha (PGC-1α) and transcriptional regulator CBP (CBP) by LXRα. No differences in co-activator recruitment were observed in the presence of vehicle (DMSO) or E_2 (5 nM), which indicates that the competition was ERα dependent but ligand independent. This further confirmed the antagonist activity of unliganded ERα reported in Figures 5A and 5C. To better evaluate the physiological significance of the two assays, we measured the relative concentrations of ERα and LXRα in the liver by qPCR; we found the results to be 1:13 at P and 1:15 at E, as the concentrations of the two mRNAs change across the estrous cycle. In the transfection and FRET assays, ERα interference was observed with a stoichiometry of at least 1:5, indicating that in the in vitro assays, the proportion between ERα and LXRα was significantly different than in liver. This led us to verify the potential for interaction of the two receptors on the promoter of the genes responsive to the presence/absence of ERα. Prior studies in liver on chromatin immunoprecipitation (ChIP) with ERα (Gao et al., 2008) and LXRα (Boergesen et al., 2012) supported the idea of a cross-coupling between the two hepatic receptors, showing that they recognized overlapping sites (see Figure S7) in the promoter/enhancer of several genes of lipid metabolism. Therefore, we carried out a series of ChIP-qPCR studies on female livers harvested at each phase of the estrous cycle. Figure 6 shows that ERα and LXRα recognize and bind the same regions of chromatin and that the reproductive cycle has a significant influence on the

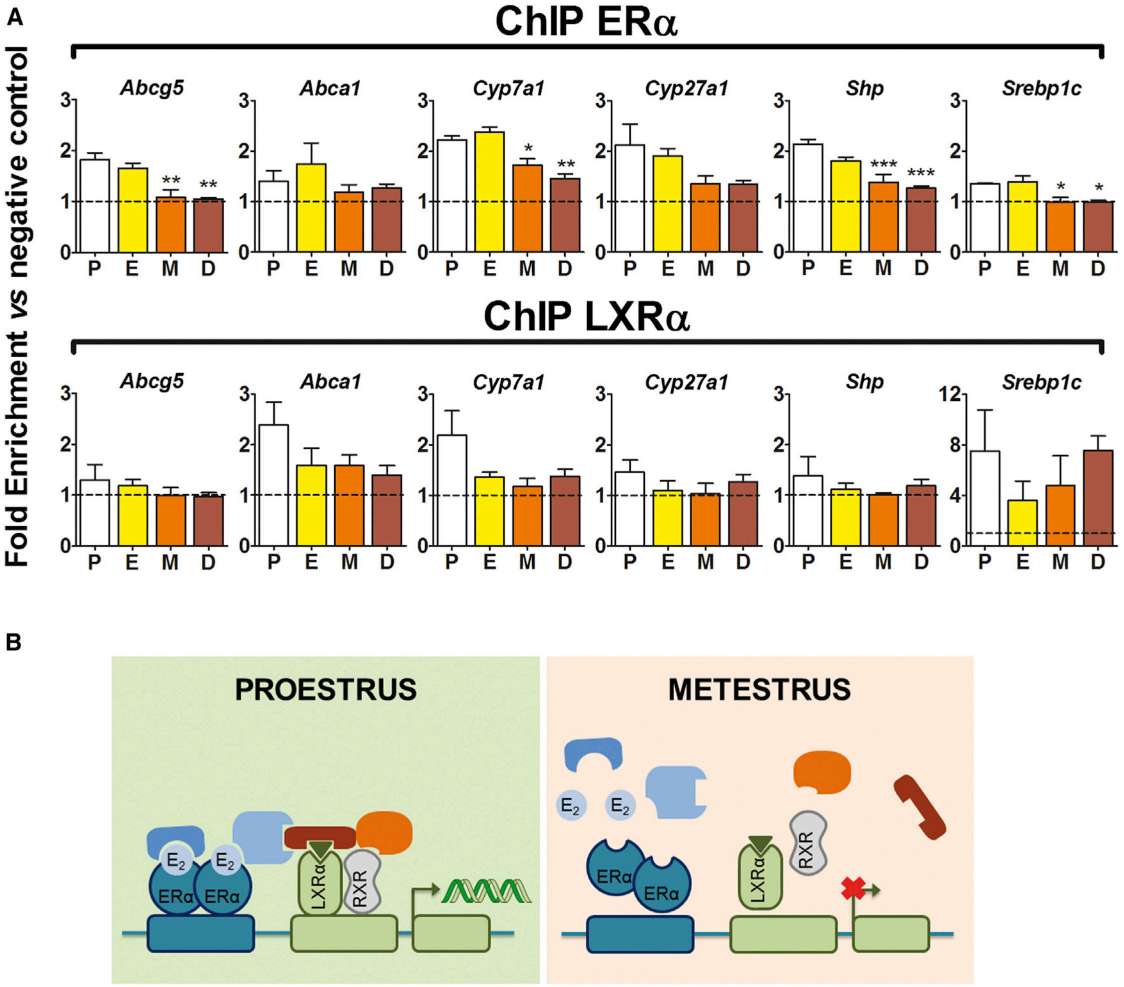

Figure 6. ERα and LXRα Functional Interaction in Liver

(A) Recruitment of ERα (upper) and LXRα (lower) by conventional ChIP followed by qPCR. ChIP was done using ERα antibody, LXRα antibody, or normal rabbit IgG as negative control. After reverse cross-link, the purified ChIP-enriched fragments were amplified using qPCR, with primers that target the selected regions (see Supplemental Experimental Procedures). For each gene, the recruitment of ERα or LXRα is expressed as ratio of the fold enrichment relative to occupancy in IgG-precipitated samples versus the FE of the negative control FoxL2: an exonic region not bound by any nuclear receptor in this cell type.

(B) ERα-LXRα cross-coupling over the course of the mouse reproductive cycle. In P, the high concentration of circulating estrogens enhances ERα binding to DNA, thereby promoting LXRα binding and transcriptional activity. In M, circulating estrogens are low, ERα binding to DNA is loosened, and LXRα binding to DNA and transcriptional activity are reduced.

The data indicate mean ± SEM. $^*p < 0.05$, $^{**}p < 0.01$, and $^{***}p < 0.001$ versus SYN at P.

extent to which both receptors interact with the promoter/enhancer of several of the genes relevant for CH metabolism. Most important is the finding that, in general, ERα maximal binding to the promoter/enhancer of the genes studied occurs at P, when estrogen production is highest; the observation that the target DNA co-precipitated with ERα at E shows that the receptor association with these promoters persists in time. For most of the promoters/enhancers studied, LXRα binding was generally higher at P, thus indicating the potential for a positive co-operation between the two hormonally regulated transcription factors. These observations led us to propose that, at the end of the follicular phase, during P, the high levels of estrogens promote the binding of ERα to the DNA in regions proximal to LXR-binding sites facilitating the transcription of LXRα target genes. The decreased concentration of estrogens loosens ERα interactions with both the DNA and LXRα: the consequence is the decreased synthesis of mRNA observed at M (Figure 6D). Consistent with the expression studies, the association of ERα with the *Srebp1-1c* promoter did not change significantly with the cycle; quite unexpected, however, was the finding that the highest fluctuation of LXRα binding across the cycle was observed on the *Srebp-1c* promoter, in spite of the fact that the amount of *Srebp-1c* mRNA is not influenced by the cycle.

DISCUSSION

The present study demonstrates that ERα is essential for coupling liver CH metabolism to ovarian cycle and for the recurrent

production of a class of HDLs uniquely suited for CH efflux; in addition, the study suggests the existence of a functional interaction between LXRα and ERα possibly designed to better adapt hepatic lipid metabolism to the needs of reproductive functions.

Liver ERα and HDL Structure and Function

Hepatocytes are the major site of CH metabolism and transport. Lipoproteins synthesized by the liver transport CH to peripheral tissues and retrieve CH from non-hepatic tissues when CH concentration is excessive through a process called "reverse CH transport" (RCT). RCT is a potent defense mechanism against CH accumulation that is performed by HDLs. The efficiency of CH efflux from peripheral cells is determined by the size, shape, and composition of the HDLs (Gursky et al., 2013). HDL protein and lipid composition is also dependent on the activity of several remodeling enzymes (Rye and Barter, 2014). These enzymes include PL transfer protein (PLTP), which mediates PL transport among HDLs and reduces the number of small HDL particles, lipases (hepatic and endothelial) that hydrolyze TG-enriched HDLs to generate small HDL particles, and CE transfer protein (CETP).

Here, we show that, in fertile females, HDLs periodically undergo a significant structural and functional remodeling that is liver ERα dependent and results in HDL reduction in size and increased ability to promote CH efflux. This effect might be caused by ERα-dependent regulation of the genes encoding (i) HDL remodeling enzymes or (ii) specific apolipoproteins more efficient in CH transport.

Indeed, we found that liver ERα was required for the differential expression of the genes encoding PLTP and hepatic lipase observed across the estrous cycle. This finding is consistent with the results of previous studies that have demonstrated that these enzymes are subject to hormonal regulation in experimental models and humans. The mechanism underlying this regulation remains to be better studied, because ERα was shown to repress or induce the transcription of the lipase gene depending on the nature, concentration, and mode of administration of the estrogenic compounds (Brinton, 1996; Tilly-Kiesi et al., 1997). The discrepancies in the literature suggest that the activity of ERα on the promoters of these genes does not occur via the direct binding to the estrogen responsive element (ERE) but rather involves the regulation of other transcription factors, such as c-*fos*, that modulate these genes via AP-1 binding sites (Jones et al., 2002).

On the other hand, the finding that P-HDLs carry a quantity of TGs twice that of the HDL isolated at E, in spite of their low dimensions and protein content (Table 1), supports the view that the P-HDLs are made of specific proteins able to bind lipids with high affinity and to reduce the size of these particles. A good candidate could be apolipoprotein M (apo-M), shown to be regulated by estrogen-activated ERα (Wei et al., 2011) and by LXRα (Nielsen et al., 2009) in several models. ApoM is primarily present in HDL, and the absence of its synthesis was associated with unusually large HDLs and the disappearance of pre-β-HDLs (highly relevant for CH efflux from macrophages).

Liver ERα and CH Transport and Uptake

This study showed that hepatic ERα is necessary for circulating estrogens to regulate a panoply of mechanisms that aim to achieve a more dynamic HDL reshaping and more efficient RCT. During P in mice expressing liver ERα, HDL synthesis (apo-E and apo-AI, and possibly others) and uptake (SR-B1 and LDLR) were increased, the transcriptions of genes that encode remodeling enzymes (*Pltp* and *Lipc*) changed, and hepatic CH transport (*Abca1* and *Abcg5*) was high; these observations indicate a role of the receptor in the coordination of these functions that is relevant to CH use and disposal.

We also observed the effects of the cycle on VLDLs and LDLs, which represent minor components of the circulating lipoproteins in mice. The CH profiles (Figure 3A) of mice with low-circulating estrogens (i.e., the mice at E and M and the OVX mice) exhibited peaks of higher molecular weights that are known to correspond to VLDL and LDL; these peaks disappeared or changed size with increases in circulating estrogens (i.e., during D and P). However, these changes were also observed in the LERKO mice, suggesting that liver ERα is not the only factor that regulates the metabolism of plasma LDL and VLDL in mice.

This strong dependence of liver ERα on the ovarian production of estrogens might easily explain why CH and lipoprotein metabolism are heavily affected by estrogen loss in OVX animals and why women, who appear to be protected against fatty liver disease/NAFLD and CVD during fertile ages, exhibit an increased incidence of these pathologies early after menopause.

Functional Interaction between LXRα and ERα

The present study shows that, in the liver, ERα is necessary to regulate LXRα activity on a selected subset of its target genes. ChIP-qPCR analyses suggest that ERα control on LXRα transcriptional activity occurs via an interaction of the two receptors on the promoter/enhancer region of LXRα target genes. The concomitant presence of ERα and LXRα occurs mainly at P, the phase of the estrous cycle where we measured the highest expression of LXRα target genes: this indicates that (1) ERα is hormone activated when co-recruited to the DNA and (2) ERα promotes LXRα transcriptional activity. This appears to be in contradiction with the transfection and FRET studies, in which we found that the unliganded ERα represses LXRα transcriptional activity or binding to co-regulators; the reason for this difference is likely ascribable to the vast excess of the ERα necessary to measure ERα effects in the in vitro studies. Thus, the in vitro studies might suggest the possibility of a variety of functional interactions between the two receptors, which can be achieved by changing receptor and ligand concentrations. This flexibility may be very relevant from the physiological point of view required to provide the degree of variability indispensable for adapting liver lipid metabolism to the needs of the reproductive systems throughout development, pregnancy, and lactation.

Prior work showed that ERα transcriptional efficiency is very sensitive to hormonal dosages, and, depending on its concentration, the same ligand can induce opposite effects (Calabrese, 2001; Li et al., 2007); this study suggests that receptor dosage is also functionally relevant and needs to be taken into consideration when analyzing ERα activities. These findings reveal how critical the interpretation is of experiments conducted with the OVX/hormone replacement paradigm, in which the ERα cell

content may be significantly changed and natural or powerful synthetic ligands are used at high doses. Indeed, using this latter paradigm, Han et al. reached conclusions opposite to ours, by showing that liver ERα represses lipid synthesis through a functional interaction with LXRα on the SREBP-1c promoter (Han et al., 2014).

Conclusions

We propose that the sequence of the events that is regulated by liver ERα across the different phases of the estrous cycle is necessary for the clearance of the excess of CH that the liver produces and transports to the periphery during specific phases of the reproductive cycle. Possibly, this mechanism was selected during evolution to ensure that the excess of CH made available under the pressure of the reproductive system was not wasted but could be efficiently re-utilized.

At the present time, in which dietary CH is excessive, the mechanism mentioned earlier has become important for health because the periodic production of highly efficient HDL might protect fertile women against the formation of undesired deposits of lipids in the peripheral tissues or blood vessels. At the same time, the tight cross-coupling between liver ERα and LXRα efficiently regulates hepatic lipid homeostasis to meet the requirements of the different reproductive stages. With the cessation of ovarian activity, this finely tuned sequence of events is disrupted, enabling the initial formation of unhealthy deposits of lipids in both the liver and periphery. Indeed, it is known that the dysregulation of CH metabolism is associated with the severity of fatty liver disease/NAFLD (Bashiri et al., 2013; Min et al., 2012) and that menopause increases the prevalence of fatty liver disease/NAFLD (Gutierrez-Grobe et al., 2010), as well as a number of associated pathologies.

Notwithstanding the differences in CH homeostasis in mice and humans (Xiangdong et al., 2011), these findings might also explain the sexually dimorphic prevalence of hepatic and cardiovascular disorders among humans under 50 years of age. Males express minimal amounts of ERα in the liver and, unlike women, do not synthesize super-efficient HDL at regular intervals. Therefore, even minimal derangements in lipid homeostasis might cause undesired accumulations of lipids over time that increase men's susceptibilities to pathologies associated with altered lipid metabolism.

Finally, the identification of liver ERα as an important factor for hepatic metabolic homeostasis underscores its importance as a primary target for post-menopausal hormone replacement therapy (HRT), because the appropriate maintenance of liver ERα activity after menopause might be the key for the prevention of disorders associated with unbalanced CH metabolism. The cyclic activation of liver ERα would reduce cardiovascular risks by promoting CH efflux from macrophages and BA synthesis without increasing fatty acid synthesis and their plasmatic levels. The use of a estrogenic compound would, therefore, be preferable over LXR agonists which have the main undesired effect of increased FA content in the liver and in the plasma (Joseph et al., 2002). Therefore, we propose the development of an appropriate HRT that targets liver ERα as a therapy of choice for the prevention of liver and cardiovascular disorders associated with the post-menopausal period.

EXPERIMENTAL PROCEDURES

Animals

The LERKO mice were obtained and maintained as previously described (Della Torre et al., 2011). Unless otherwise stated, the mice were 3 months of age. Vaginal smears were performed at 9:00 a.m. To avoid any possible confounding effect due to the circadian rhythm or feeding status, the mice were euthanized after 6 hr of fasting between 2:00 and 4:00 p.m. (Della Torre et al., 2011). All animal experimentation was performed in accordance with the ARRIVE guidelines and the European guidelines for animal care and the use of experimental animals, approved by the Italian Ministry of Research and University, and controlled by a departmental panel of experts.

Liver Histology

See Supplemental Experimental Procedures.

FPLC Analyses

The CH distribution in the plasma lipoprotein fractions was determined via FPLC using a Superose 6 column (Amersham Biosciences). 500-μl fractions were collected and assayed for CH with an enzymatic kit (Sentinel).

HDL Purification, Composition, and Size

HDLs (d = 1.063–1.21 g/ml) were purified from pooled plasma samples by sequential ultracentrifugation, using a TL100.3 rotor in a TL100 ultracentrifuge (Beckman Coulter). The total and unesterified CH (TC and UC, respectively), TG, and PL contents of the isolated lipoproteins were measured by standard enzymatic techniques. The CE mass was calculated as (TC − UC) × 1.68. The protein content was assessed by the Lowry method. The HDL composition was calculated as the percentage of the particle total mass. HDL particle sizes were analyzed by non-denaturing polyacrylamide GGE using precast 4%–30% acrylamide gels (CBS Scientific). Coomassie-stained gels were scanned with a GS-690 densitometer, and particle sizes were calculated with the Multi-Analyst software (Bio-Rad).

CEC

See Supplemental Experimental Procedures.

Biochemical Assays

The CH, CE, FFA, and Tg levels in the liver tissues were measured with appropriate kits according to the manufacturer's protocols (Biovision).

Fecal BA Excretion

Dried feces were extracted in 1 ml of 75% ethanol at 50°C for 2 hr. The extracts were centrifuged, and the supernatants were diluted 20-fold with 65 mM phosphate buffer at pH 7.0. BA concentration was measured with an enzymatic kit (Sentinel).

Western Blot Analysis

See Supplemental Experimental Procedures.

Real-Time PCR Gene Expression Analysis

Total liver RNA extraction, cDNA synthesis, real-time PCR, and data analysis were performed as previously described (Della Torre et al., 2011). The primers used are listed in the Supplemental Experimental Procedures.

Cell Cultures and Transfections

See Supplemental Experimental Procedures.

Co-regulator Recruitment by FRET Assays

See Supplemental Experimental Procedures.

ChIP-qPCR Experiments

ChIP-qPCR experiments were conducted as previously described (Uhlenhaut et al., 2013): formaldehyde-fixed mouse liver chromatin was processed for ChIP using the following antibodies: ERα (sc-542, Santa Cruz Biotechnology) and LXRα (pp-pp70412-00, R&D Systems); and SYBR Green qPCR was performed on a ViiA 7 instrument (Applied Biosystems).

The primer sequences are listed in the Supplemental Experimental Procedures.

Statistical Analyses

Unless otherwise stated, statistical significance was assessed by one-way or two-way ANOVA using Bonferroni's multiple comparison post hoc tests that were performed with GraphPad Prism 5 (GraphPad Software). *p < 0.05, **p < 0.01 and ***p < 0.001 versus SYN at P; ^{O}p < 0.05, ^{OO}p < 0.01 and ^{OOO}p < 0.001 versus LERKO at P; #p < 0.05, ##p < 0.01 and ###p < 0.001 versus SYN.

SUPPLEMENTAL INFORMATION

Supplemental Information includes Supplemental Experimental Procedures, seven figures, and one table and can be found with this article online at http://dx.doi.org/10.1016/j.celrep.2016.03.019.

AUTHOR CONTRIBUTIONS

S.D.T. designed the project and conducted most of the in vivo studies, biochemical assays, and ChIP studies; S.D.T. wrote and revised the manuscript. N.M. conceived and conducted the CH-related analysis and the FRET experiments. R.F. performed the co-transfection studies. M.G. performed the lipoprotein experiments. E.F. performed the CEC analysis. C.R. performed the anatomo-pathological analysis of liver tissues. F.L. performed the real-time PCR experiments. F.Q. collaborated in the ChIP studies. C.M. performed the immunostaining experiments. C.O. measured plasma 17β-estradiol. M.C. participated in the discussion of the results and revised the manuscript. N.H.U. conceived the ChIP studies and revised the manuscript. L.C. conceived the lipoprotein experiments and revised the manuscript. A.M. conceived the project, wrote the manuscript, and supervised the entire project.

ACKNOWLEDGMENTS

This paper is dedicated to the memory of our veterinarian, dear friend, and extraordinary gentleman, Dr. Paolo Sparaciari. We are grateful to Dr. J.W. Schwabe and Dr. D. Moras for providing the LXRα and ERα expression vectors; Dr. D.J. Mangelsdorf for providing the ABCA1 and SREBP-1c luciferase reporter plasmids; and Francesca Zimetti for the CEC assays. This work was supported by a grant from the European Community (ERC-Advanced Grant Ways 322977) and by Cariplo Foundation (Grant 2013-0786).

Received: October 27, 2015
Revised: December 21, 2015
Accepted: March 2, 2016
Published: March 31, 2016

REFERENCES

Barsalani, R., Chapados, N.A., and Lavoie, J.M. (2010). Hepatic VLDL-TG production and MTP gene expression are decreased in ovariectomized rats: effects of exercise training. Horm. Metab. Res. *42*, 860–867.

Bashiri, A., Tavallaee, G., Li, L., and Ng, D.S. (2013). Emerging role of cellular cholesterol in the pathogenesis of nonalcoholic fatty liver disease. Curr. Opin. Lipidol. *24*, 275–276.

Bocher, V., Pineda-Torra, I., Fruchart, J.C., and Staels, B. (2002). PPARs: transcription factors controlling lipid and lipoprotein metabolism. Ann. N Y Acad. Sci. *967*, 7–18.

Boergesen, M., Pedersen, T.A., Gross, B., van Heeringen, S.J., Hagenbeek, D., Bindesbøll, C., Caron, S., Lalloyer, F., Steffensen, K.R., Nebb, H.I., et al. (2012). Genome-wide profiling of liver X receptor, retinoid X receptor, and peroxisome proliferator-activated receptor α in mouse liver reveals extensive sharing of binding sites. Mol. Cell. Biol. *32*, 852–867.

Brinton, E.A. (1996). Oral estrogen replacement therapy in postmenopausal women selectively raises levels and production rates of lipoprotein A-I and lowers hepatic lipase activity without lowering the fractional catabolic rate. Arterioscler. Thromb. Vasc. Biol. *16*, 431–440.

Calabrese, E.J. (2001). Estrogen and related compounds: biphasic dose responses. Crit. Rev. Toxicol. *31*, 503–515.

Ciana, P., Raviscioni, M., Mussi, P., Vegeto, E., Que, I., Parker, M.G., Lowik, C., and Maggi, A. (2003). In vivo imaging of transcriptionally active estrogen receptors. Nat. Med. *9*, 82–86.

Ciana, P., Brena, A., Sparaciari, P., Bonetti, E., Di Lorenzo, D., and Maggi, A. (2005). Estrogenic activities in rodent estrogen-free diets. Endocrinology *146*, 5144–5150.

Clegg, D.J. (2012). Minireview: the year in review of estrogen regulation of metabolism. Mol. Endocrinol. *26*, 1957–1960.

Della Torre, S., Rando, G., Meda, C., Stell, A., Chambon, P., Krust, A., Ibarra, C., Magni, P., Ciana, P., and Maggi, A. (2011). Amino acid-dependent activation of liver estrogen receptor alpha integrates metabolic and reproductive functions via IGF-1. Cell Metab. *13*, 205–214.

Della Torre, S., Benedusi, V., Fontana, R., and Maggi, A. (2014). Energy metabolism and fertility: a balance preserved for female health. Nat. Rev. Endocrinol. *10*, 13–23.

Favari, E., Ronda, N., Adorni, M.P., Zimetti, F., Salvi, P., Manfredini, M., Bernini, F., Borghi, C., and Cicero, A.F. (2013). ABCA1-dependent serum cholesterol efflux capacity inversely correlates with pulse wave velocity in healthy subjects. J. Lipid Res. *54*, 238–243.

Gambarin-Gelwan, M., Kinkhabwala, S.V., Schiano, T.D., Bodian, C., Yeh, H.C., and Futterweit, W. (2007). Prevalence of nonalcoholic fatty liver disease in women with polycystic ovary syndrome. Clin. Gastroenterol. Hepatol. *5*, 496–501.

Gao, H., Fält, S., Sandelin, A., Gustafsson, J.A., and Dahlman-Wright, K. (2008). Genome-wide identification of estrogen receptor alpha-binding sites in mouse liver. Mol. Endocrinol. *22*, 10–22.

Gursky, O., Jones, M.K., Mei, X., Segrest, J.P., and Atkinson, D. (2013). Structural basis for distinct functions of the naturally occurring Cys mutants of human apolipoprotein A-I. J. Lipid Res. *54*, 3244–3257.

Gutierrez-Grobe, Y., Ponciano-Rodríguez, G., Ramos, M.H., Uribe, M., and Méndez-Sánchez, N. (2010). Prevalence of non alcoholic fatty liver disease in premenopausal, posmenopausal and polycystic ovary syndrome women. The role of estrogens. Ann. Hepatol. *9*, 402–409.

Han, S.I., Komatsu, Y., Murayama, A., Steffensen, K.R., Nakagawa, Y., Nakajima, Y., Suzuki, M., Oie, S., Parini, P., Vedin, L.L., et al. (2014). Estrogen receptor ligands ameliorate fatty liver through a nonclassical estrogen receptor/Liver X receptor pathway in mice. Hepatology *59*, 1791–1802.

Jones, D.R., Schmidt, R.J., Pickard, R.T., Foxworthy, P.S., and Eacho, P.I. (2002). Estrogen receptor-mediated repression of human hepatic lipase gene transcription. J. Lipid Res. *43*, 383–391.

Joseph, S.B., Laffitte, B.A., Patel, P.H., Watson, M.A., Matsukuma, K.E., Walczak, R., Collins, J.L., Osborne, T.F., and Tontonoz, P. (2002). Direct and indirect mechanisms for regulation of fatty acid synthase gene expression by liver X receptors. J. Biol. Chem. *277*, 11019–11025.

Li, A.C., and Glass, C.K. (2004). PPAR- and LXR-dependent pathways controlling lipid metabolism and the development of atherosclerosis. J. Lipid Res. *45*, 2161–2173.

Li, L., Andersen, M.E., Heber, S., and Zhang, Q. (2007). Non-monotonic dose-response relationship in steroid hormone receptor-mediated gene expression. J. Mol. Endocrinol. *38*, 569–585.

Min, H.K., Kapoor, A., Fuchs, M., Mirshahi, F., Zhou, H., Maher, J., Kellum, J., Warnick, R., Contos, M.J., and Sanyal, A.J. (2012). Increased hepatic synthesis and dysregulation of cholesterol metabolism is associated with the severity of nonalcoholic fatty liver disease. Cell Metab. *15*, 665–674.

Nielsen, L.B., Christoffersen, C., Ahnström, J., and Dahlbäck, B. (2009). ApoM: gene regulation and effects on HDL metabolism. Trends Endocrinol. Metab. *20*, 66–71.

Ostberg, J.E., Thomas, E.L., Hamilton, G., Attar, M.J., Bell, J.D., and Conway, G.S. (2005). Excess visceral and hepatic adipose tissue in Turner syndrome

determined by magnetic resonance imaging: estrogen deficiency associated with hepatic adipose content. J. Clin. Endocrinol. Metab. *90*, 2631–2635.

Roeters van Lennep, J.E., Westerveld, H.T., Erkelens, D.W., and van der Wall, E.E. (2002). Risk factors for coronary heart disease: implications of gender. Cardiovasc. Res. *53*, 538–549.

Rohatgi, A., Khera, A., Berry, J.D., Givens, E.G., Ayers, C.R., Wedin, K.E., Neeland, I.J., Yuhanna, I.S., Rader, D.R., de Lemos, J.A., and Shaul, P.W. (2014). HDL cholesterol efflux capacity and incident cardiovascular events. N. Engl. J. Med. *371*, 2383–2393.

Rye, K.A., and Barter, P.J. (2014). Regulation of high-density lipoprotein metabolism. Circ. Res. *114*, 143–156.

Tilly-Kiesi, M., Kahri, J., Pyörälä, T., Puolakka, J., Luotola, H., Lappi, M., Lahdenperä, S., and Taskinen, M.R. (1997). Responses of HDL subclasses, Lp(A-I) and Lp(A-I:A-II) levels and lipolytic enzyme activities to continuous oral estrogen-progestin and transdermal estrogen with cyclic progestin regimens in postmenopausal women. Atherosclerosis *129*, 249–259.

Uhlenhaut, N.H., Barish, G.D., Yu, R.T., Downes, M., Karunasiri, M., Liddle, C., Schwalie, P., Hübner, N., and Evans, R.M. (2013). Insights into negative regulation by the glucocorticoid receptor from genome-wide profiling of inflammatory cistromes. Mol. Cell *49*, 158–171.

Wei, J., Shi, Y., Zhang, X., Feng, Y., Luo, G., Zhang, J., Mu, Q., Tang, Y., Yu, Y., Pan, L., et al. (2011). Estrogen upregulates hepatic apolipoprotein M expression via the estrogen receptor. Biochim. Biophys. Acta *1811*, 1146–1151.

Xiangdong, L., Yuanwu, L., Hua, Z., Liming, R., Qiuyan, L., and Ning, L. (2011). Animal models for the atherosclerosis research: a review. Protein Cell *2*, 189–201.

Zhang, Y., Breevoort, S.R., Angdisen, J., Fu, M., Schmidt, D.R., Holmstrom, S.R., Kliewer, S.A., Mangelsdorf, D.J., and Schulman, I.G. (2012). Liver LXRα expression is crucial for whole body cholesterol homeostasis and reverse cholesterol transport in mice. J. Clin. Invest. *122*, 1688–1699.

Cell Reports, Volume *15*

Supplemental Information

An Essential Role for Liver ERα in Coupling Hepatic Metabolism to the Reproductive Cycle

Sara Della Torre, Nico Mitro, Roberta Fontana, Monica Gomaraschi, Elda Favari, Camilla Recordati, Federica Lolli, Fabiana Quagliarini, Clara Meda, Claes Ohlsson, Maurizio Crestani, Nina Henriette Uhlenhaut, Laura Calabresi, and Adriana Maggi

Figure S1, related to Figure 1

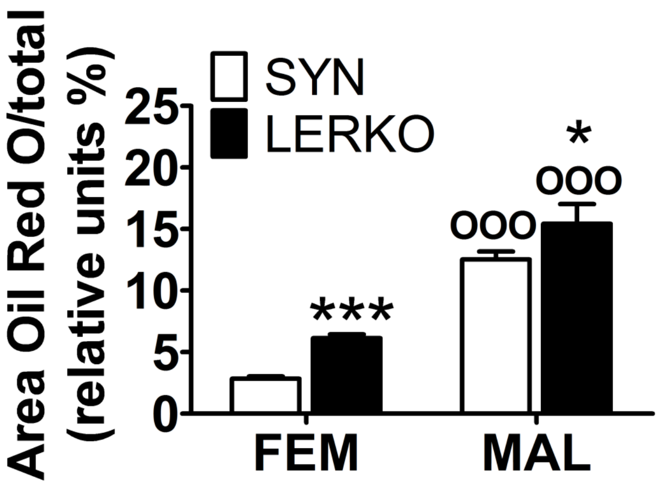

Figure S1 (related to Figure 1). Liver fat deposition in SYN and LERKO female and male mice.

Quantification of the lipid deposits by Oil Red O staining in the liver tissues of SYN and LERKO female (FEM) and male (MAL) mice of 10 months of age. The data are expressed as percentages of the total section areas. The bars are mean ± SEM, n=6; the experiment was repeated 3 times. **p<0.01 and ***p<0.001 *versus* SYN; ^{OOO}p<0.001 *versus* females by two-way ANOVA followed by Bonferroni *post-hoc* test.

Figure S2, related to Figure 1

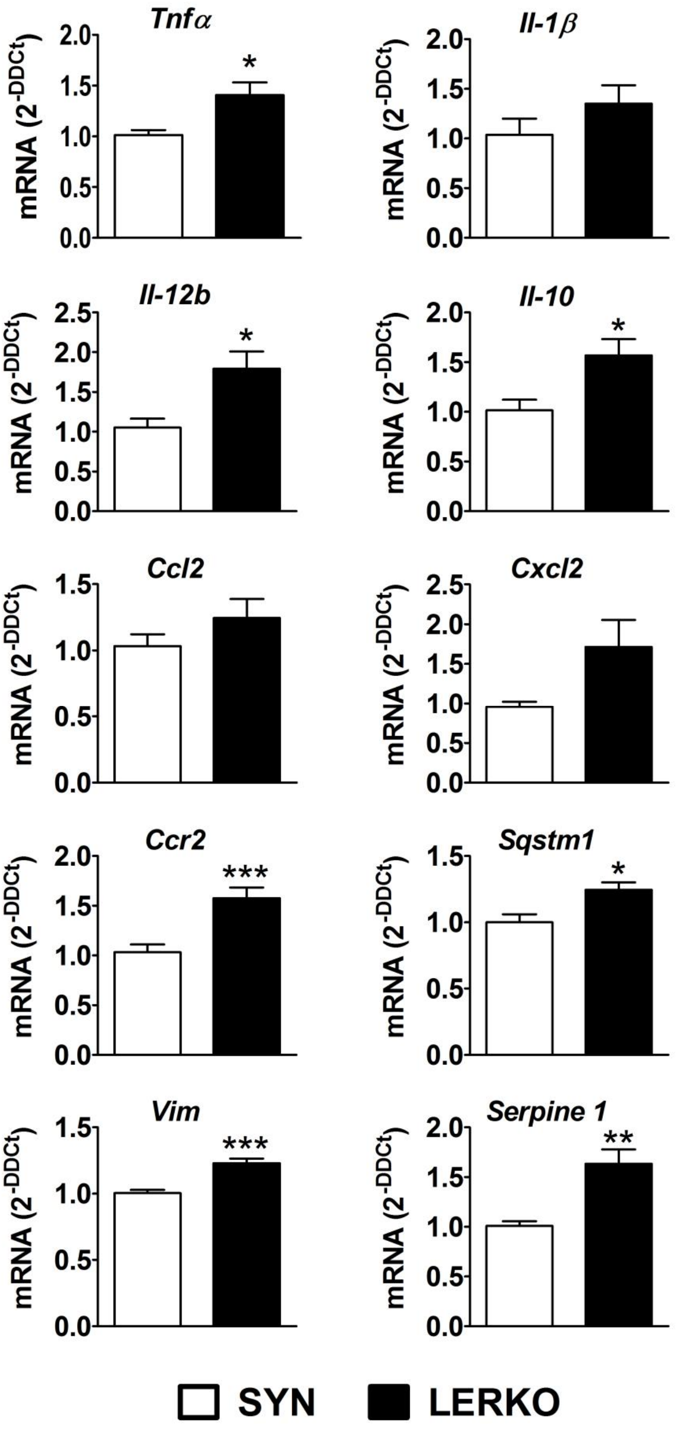

Figure S2 (related to Figure 1). **The contents of inflammatory marker mRNAs in the liver tissues of SYN and LERKO females (at metestrus) at 10 months of age**. The expression of several genes involved in the inflammatory process (tumor necrosis factor alpha, *TnfŬ*; interleukin-1 beta, *Il-1ɓ*; interleukin-12 beta; *Il-12ɓ*; interleukin-10, *Il-10*; chemokine (C-C motif) ligand 2, *Ccl2*; chemokine (C-X-C motif) ligand 2, *Cxcl2*; chemokine (C-C motif) receptor 2, *Ccr2*) and collagen deposition (sequestosome1, *Sqstm1*; vimentin, *Vim*; serpine1, *Serpine*) was measured by rtPCR. The bars are mean ± SEM, n=15. *p<0.05 and **p<0.01 *versus* SYN by t-test.

Figure S3, related to Figure 2

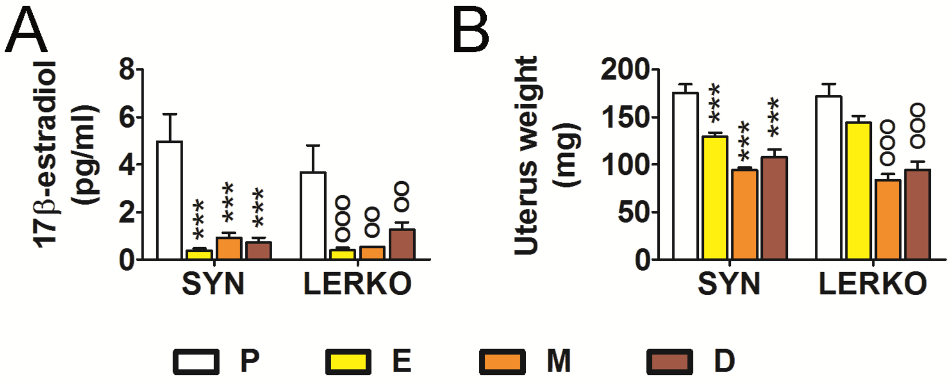

Figure S3 (related to Figure 2). Circulating 17β-estradiol levels and uterine weight changes in SYN and LERKO females during the estrous cycle progression. A. 17β-estradiol levels measured in the plasma of SYN and LERKO females during the estrous cycle. The bars are mean ± SEM, n=4. ***p<0.001 *versus* SYN at P; ^{OO}p<0.01 and ^{OOO}p<0.001 *versus* LERKO at P by two-way ANOVA followed by Bonferroni *post-hoc* test. **B.** Uterine weight changes in SYN and LERKO females during the estrous cycle progression. The bars are mean ± SEM, n=6; the experiment was repeated 3 times. ***p<0.001 *versus* SYN at P; ^{OOO}p<0.001 *versus* LERKO at P by two-way ANOVA followed by Bonferroni *post-hoc* test.

Figure S4, related to Figure 3

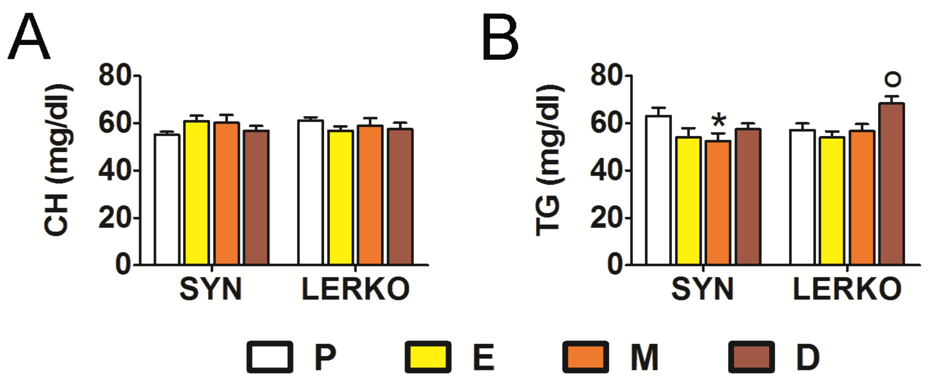

Figure S4 (related to Figure 3). Total cholesterol and triglycerides in the plasmas of SYN and LERKO females during the estrous cycle. The bars are mean ± SEM, n=6; the experiment was repeated 3 times. *$p<0.05$ *versus* SYN at P; $^{O}p<0.05$ *versus* LERKO at P by two-way ANOVA followed by Bonferroni *post-hoc* test.

Figure S5, related to Figure 3A

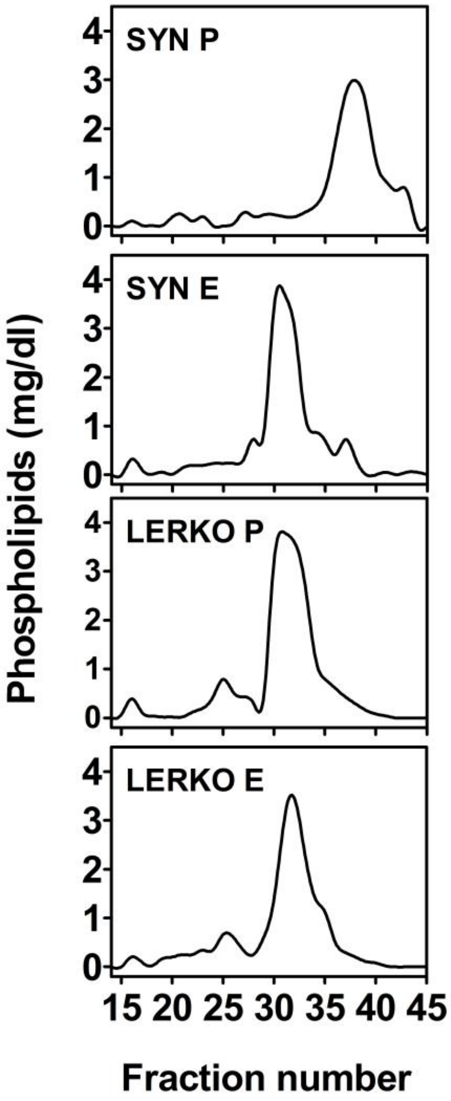

Figure S5 (related to Figure 3A). **Phospholipid profiles of the FPLC-fractioned plasma samples from the SYN and LERKO females.** The graph shows the phospholipid contents in mg/dl that were measured in FPLC-fractioned plasmas of the SYN and LERKO females at proestrus (P) and estrus (E).

Figure S6, related to Figure 3

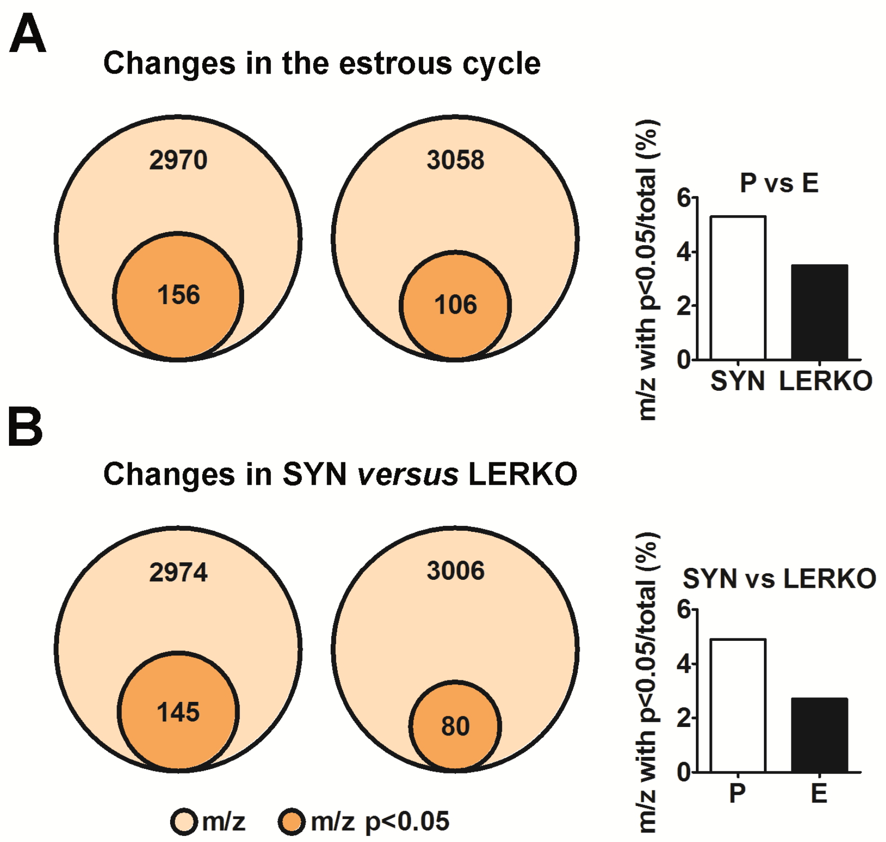

Figure S6 (related to Figure 3). **Plasma metabolomic analyses of the SYN and LERKO females. A**. The pie charts represent the total number of metabolites (light orange) and the number of metabolites that were differentially expressed (dark orange) between P and E in the SYN and LERKO mice. The graph represents the number of metabolites differentially modulated between P and E expressed as a percentage respect the total number of metabolites that were identified in the plasmas of the SYN and LERKO females. **B**. The pie chart represents the total number of metabolites (light orange) and the number of metabolites that were differentially expressed (dark orange) at P and E in the SYN and LERKO mice. The graph represents the number of metabolites that were differentially regulated between the SYN and LERKO mice expressed as percentages of the total number of metabolites identified in the plasmas of the females at P and E, respectively.

Figure S7, related to Figure 6

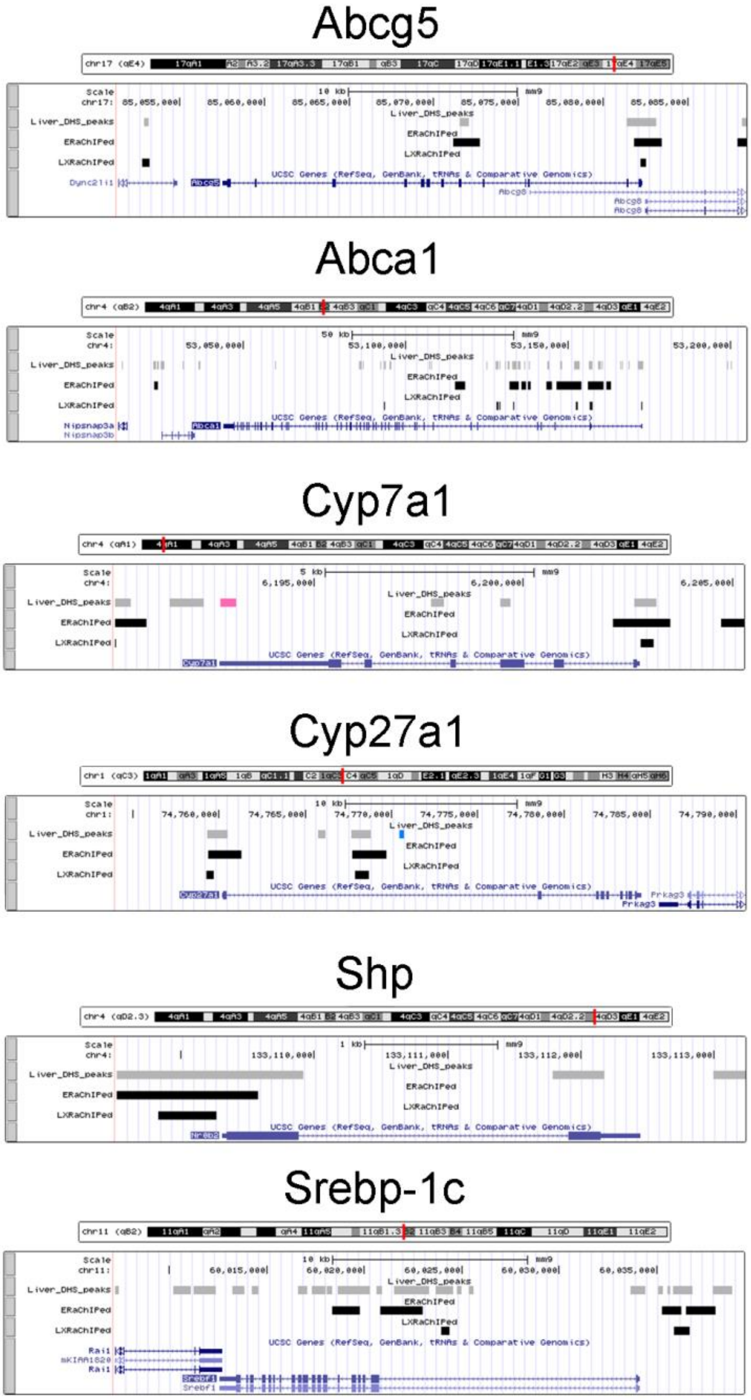

Figure S7 (related to Figure 6). **UCSC Genome Browser tracks derived from ChIP-seq data showing ERŬ and LXRŬ binding to the *Abcg5*, *Abca1*, *Cyp7a1*, *Cyp27a1*, *Shp*, and *Srebp-1c***. The black bars indicate binding of ERŬ (labeled as ñUser Track: ERŬChIPedò) and LXRŬ (labeled as ñUser Track: LXRŬChIPedò). The gray bars show the liver DHS peaks identified by analyzing the genome-wide DNase hypersensitivity in mouse liver mapped by DNase I treatment coupled with high-throughput sequencing (DNase-seq) (Rozowsky et al., 2009).

Table S1

	Females SYN	Females LERKO
Hepatocellular vacuolar degeneration (fatty change)	++	++
Droplet size	+	++/+++
Portal inflammation	+	++
Inflammatory foci	-	+
Portal fibrosis	-	-
Portal and/or centrilobular fibrosis	-	+/++

Table S1. **Anatomo-pathological analysis of the livers of SYN and LERKO female mice at 10 months of age.** Liver sections were routinely stained with hematoxylin and eosin (H&E) and evaluated in a blinded fashion under a light microscope. A semiquantitative grading of hepatocellular vacuolar degeneration and portal inflammation (i.e., the infiltration of mononuclear inflammatory cells) was performed.

SUPPLEMENTAL EXPERIMENTAL PROCEDURES

Liver histology: The left lobe of the liver was fixed in 10% neutral formalin solution (Sigma-Aldrich) overnight at 4°C, cryopreserved in a 30% (w/v) sucrose solution for 24 hours at 4°C and stored at -80°C. 7 µm-thick liver sections were cut with a refrigerated microtome (Leica), collected on slides and stored at -80°C until staining. Hematoxylinïeosin (H&E) staining was performed on the frozen slides with Mayer hematoxylin (Bio-Optica) for 1 min and, after washing with water, with 1% eosin aqueous solution (Bio-Optica) for 4 minutes. Oil Red O staining was performed as previously described (Villa et al., 2012). The Accustain Trichrome stain kit was used for Massonôs trichrome staining (Sigma-Aldrich). After staining, the slides were cleared in xylenes and cover slipped with xylenes-based mounting medium (Eukitt, Bio-Optica). The liver sections were evaluated in a blinded fashion under a light microscope. Semi-quantitative grading of the hepatocellular vacuolar degeneration and portal inflammation (i.e., the infiltration of mononuclear inflammatory cells) was performed in a blinded fashion. Images of the stained sections were captured using Microscope Axioscop2 mot plus (Zeiss).

Real-Time PCR Gene Expression Analysis: The primers used for the rtPCR reactions were as follows: *Esr1* (Mm00433147_m1), *Pltp* (Mm01240573_m1), *Lipc* (Mm01171487_m1), *PpparŬ* (Mm00440939_m1), *Cpt1Ŭ* (Mm01231183_m1), *HadhŬ* (Mm00805228_m1), *Acox* (Mm01246831_m1), *Acsl1* (Mm00484217_m1), *LxrŬ* (Mm00443451_m1), *Abcg55* (Mm00446241_m1), *Abca1* (Mm00442646_m1), *Cyp7a1* (Mm00484150_m1), *Cyp27a1* (Mm00470430_m1), *Fasn* (Mm00662319_m1), *TnfŬ* (Mm00443258_m1), *Il-1ɓ* (Mm00434228_m1), *Il-12ɓ* (Mm00434174_m1), *Il-10* (Mm00439614_m1), *Ccl2* (Mm00441242_m1), *Cxcl2* (Mm00436450_m1), *Ccr2* (Mm00438270_m1), *Sqstm1* (Mm00448091_m1), *Vim* (Mm01333430_m1), *Serpine* (Mm00435860_m1), *Shp* (Mm00442278_m1) (all from Life Technologies), and *Srebp-1c* (forward: 5´-ATGGATTGCACATTTGAAGACATGCT-3´ and reverse: 5´-CCTGTGTCCCCTGTCTCAC-3´). The *36b4* primer was been used as reference gene assay (forward: 5´-GGCGACCTGGAAGTCCAACT-3´ and reverse: 5´-CCATCAGCACCACAGCCTTC-3´).

17ɓ-estradiol levels in the plasma: Plasma levels of estradiol were measured in a single run by GC-MS/MS. Briefly, after addition of isotope-labeled standards, estradiol was extracted to chlorobutane, purified on a silica column and derivatized using pentafluorobenzyl hydroxylamine hydrochloride followed by pentafluorobenzoyl chloride. Estradiol was analyzed in multiple reaction monitoring mode with ammonia as reagent gas using an Agilent 7000 triple quadrupole mass spectrometer equipped with a chemical ionization source. The limits of quantification (LOQ) for estradiol was 0.5 pg/ml. The intra-assay coefficients of variation (CVs) were below 14% and the inter-assay CVs below 11%.

Western blot analysis: Samples of frozen mouse liver were homogenized in ice-cold buffer (20 mM HEPES, 5 mM $MgCl_2$, 420 mM NaCl, 0.1 mM EDTA, and 20% glycerol) containing protease and phosphatase inhibitors according to the manufacturerôs protocols (Phosphatase and Protease Inhibitor Mini Tablets, Pierce). After 3 repeated cycles of freezing and thawing, the homogenate was centrifuged at 16100g for 15 min at 4°C, and the supernatant was collected in a new tube. After appropriate quantitative analysis (Bradford assay, Pierce), equal amounts of the protein samples (25 µg of liver extracts or 30 µL of plasma FPLC fractions) were resuspended in Laemmli sample buffer and separated in an 8-16% SDS polyacrylamide gel system (Biorad). After transfer, the nitrocellulose membranes were incubated with specific antibodies overnight at 4°C and then with the secondary antibody conjugated with peroxidase for 1h at r.t. The primary antibodies used were the following: anti-apoAI (Rockland Immunochemicals), anti-apoE (Calbiochem), anti-LDLR and anti-SR-B1 (both Novus Biological), anti-ERŬ and anti-PPARŬ (Santa Cruz), anti-LXRŬ (R&D systems), and anti-ɓ-actin (Sigma). Immunoreactivity was detected with an ECL Western Blotting Analysis System (Amersham) and acquired and analyzed using an Odissey Fc Imaging system and the Image StudioÊ software (LiCorBiosciences).

Total cholesterol and triglyceride levels in the plasma: The CH and TG plasma levels were determined using enzymatic kits (Sentinel).

Plasma phospholipids profile: The phospholipid distributions in the FPLC-fractioned plasma were determined using an enzymatic kit (BL chimica).

Cholesterol Efflux Capacity (CEC): The CEC was evaluated with a radioisotopic assay that quantified the total cholesterol efflux mediated by pathways of known relevance in macrophages, i.e., ATP binding cassette transporter A1 (ABCA1) (Khera et al., 2011). J774 cells derived from a murine macrophage cell line were plated and radiolabeled for 24h with 2 εCi/mL of ^{3}H-cholesterol. ABCA1 was up-regulated by an 18-hour incubation with 0.3 mM of 8-(4-chlorophenylthio)-cyclic AMP (cAMP) (Favari et al., 2004b). Subsequently, the efflux medium containing 0.5% whole plasma from either SYN or LERKO female mice in either the P or E phase was added for 4 hours (to prevent serum lipoprotein remodeling at room temperature, all plasma aliquots were defrosted in ice immediately before use). All steps

were performed in the presence of 2 εg/ml of CP113,818 (an acylї coenzyme A:cholesterol acyl-transferase inhibitor) to ensure all cholesterol was in the free form (Zimetti et al., 2006). Liquid scintillation counting was used to quantify the efflux of radioactive cholesterol from the cells. The total radioactive cholesterol incorporated by cells was calculated by isopropanol extraction of control wells that were not exposed to plasma. The CEC is expressed as the percentage of the radioactivity released to the medium over the total radioactivity incorporated by the cells. To minimize the intra-assay variability, every sample was run in triplicate, and the average values and standard deviations were calculated for each percentage of efflux obtained (Favari et al., 2013). cAMP-induced ABCA1 expression was verified by the increase in efflux in response to 10 µg/mL of apoA-I that was used as an ABCA1-specific extracellular acceptor (Favari et al., 2004a). A pool of plasma was used as a reference standard to correct for the inter-assay and intra-assay variabilities (Adorni et al., 2012). The calculated mean intra- and inter-assay coefficients of variation were <6% and <4%, respectively.

Metabolomic analysis: For the LC-MS analysis, 45 εL of plasma was spiked with 5 εL of internal standard (Reserpine) at a known concentration (10 ng/mL) to control the reproducibility of the sample preparation. 100 εL of CH_3CN were added to the spiked plasma to precipitate proteins, and the sample was centrifuged. 120 εL of supernatant was collected and spiked with 100 εL of Bruker Tune mix solution (Bruker Daltonics) to check the LC-MS signal stability during the analysis. The samples were dried with a Thermo Fisher SpeedVac and resuspended in 50 εL of H_2O/CH_3OH (1:1). Before mass spectrometric analysis, analyte separation was achieved with an Ultimate 3000 UPLC (Thermo Fisher) liquid chromatography apparatus using a PFP 50x2.1 1.9µm chromatographic column (Thermo Fisher); the mobile phases were as follows: A) H_2O + 0.2% HCOOH, and B) CH_3OH. A binary gradient was used; 2% of B was maintained for 2 minutes, at 3 minutes, the B% was raised to 30. After an additional 5 minutes, the B% was brought up to 80%, and this % was maintained for 4 minutes; 2% B was reached in 1 minute, and the column was re-equilibrated in the initial conditions for 2 minutes. The chromatographic flow was 0.55 mL/min. The injection volume was 10 µL. Instrument analysis was performed with a QTOF mass analyzer (Bruker Daltonics) coupled to an ESI source and operated in positive ion mode. Full-scan spectra were acquired over the range of 100-700 m/z. The following ion source parameters were used: ESI capillary voltage, 3000 volts; dry gas flow rate, 2 L/min; Dry gas temperature, 300°C; and nebulizer gas flow rate, 80 psi. The data were converted to the mzXML format (Pedrioli et al., 2004) prior to data elaboration analysis using the Bruker Daltonics Data Analysis software. A XCMS algorithm (Smith et al., 2006) was employed in conjunction with an R statistical elaboration package (Bates D, 2014) to obtain both m/z discriminant p values (t test) and to perform the cluster analysis.

Cell cultures and transfections: HeLa cells were co-transfected using Lipofectamine LTX and Plus Reagent (Life Technologies) with 8 ng of the LXRŬ cDNA plasmids (pCMXhLXRalpha) 400 ng of the luciferase reporter (ptk3xLXRE), or ABCA1 luciferase reporter (puc18-mABCA1-pro-luc) or SREBP-1c luciferase reporter (SREBP1C-3000-luc), and 40 ng of б-galactosidase (pCMVbetaGal) in the absence/presence of increasing (ranging from 0.1 to 6.4 ng) concentrations of ERŬ (pCMVhERŬ). HepG2 cells were co-transfected by using Lipofectamine LTX and Plus Reagent with 16 ng of the plasmids coding for PPARŬ (pSG5plHPPARŬWT), 800 ng of the plasmids coding for PPAR luciferase reporter (pMAR(PPRE)5xTkLUC-MAR) and 40 ng of the plasmid pCMVbetaGal in the absence/presence of increasing concentrations of ERŬ (0.8-1.6-3.2-6.4 ng). The cells were then treated with vehicle or with the respective agonists (10 µM T0901317 for LXRŬ and 10 µM WY-14,643 for PPARŬ) in the absence/presence of 10 nM E_2 and of 1 µM ICI 182,780 (Sigma-Aldrich). After 24 hours, the cells were harvested, and luciferase enzymatic activity was measured with a luminometer (Glomax, Promega). The data were normalized to the total protein content (measured by the BCA method to avoid interfering effects) and to б-galactosidase for the transcription efficiency analyses. The data are expressed as RLU/µg proteins.

Co-regulator Recruitment by fluorescence resonance energy transfer (FRET) Assays: The ligand-binding domains (LBDs) of human LXRŬ and ERŬ were expressed as His-tagged proteins using the pET30 and pET15b vectors, respectively. Briefly, freshly transformed *E. coli* BL21 DE3 were grown in 2 L2xTY medium with 30 εg/mL of kanamycin for LXRŬ expression and 100 εg/mL of ampicillin for ERŬ expression at 37°C to an OD of 0.6-0.8. The cultures were then induced with 0.5 mM isopropyl-б-D-thiogalactopyranoside (IPTG) and further incubated at 20°C for 20h. The cells were harvested and resuspended in 40 mL of lysis buffer (50 mM Tris-HCl pH 8, 500 mM NaCl, 1% Triton X-100, 0.1% б-mercaptoethanol, 10% glycerol and protease inhibitors). The cells were sonicated, and the soluble fractions were isolated by centrifugation (35000g at 4°C for 20 min). The supernatants were incubated with Ni^{+2}-nitrilo-triacetic acid slurry for 60 min at 4°C. The slurries were washed, and the protein was recovered in 2 mL of elution buffer (50 mM Tris-HCl pH 8, 150 mM NaCl, 250 mM imidazole, 1 mM TCEP and 10%glycerol). The proteins were then dialyzed over the same buffer without imidazole, quantified and purity on SDS-PAGE.

Fluorescence energy transfer assays were performed to evaluate peptide recruitment in 384-well plates in a final volume of 10 εL. A mix of 8 ng of human LXRŬ or ERŬ LBD, 0.8 ng of Europium-labeled anti-His antibody (Perkin Elmer) and 100 ng allophycocyanin (APC)-labeled streptavidin (Perkin Elmer) in a FRET buffer containing 50 mM Tris pH 7.5, 50 mM KCl, 1 mM DTT and 0.1% free fatty acid BSA was prepared. T0901317 and 17б-estradiol were added to the mix at concentrations that ensured the saturation of the receptors. The biotinylated peptides were added in 12-point

dose response curves starting at 7 εM. The reactions were equilibrated for 1 hour at room temperature and then measured in an Envision multi-plate reader using excitation and emission wavelengths of 340 and 615 nm, respectively. The ratio between 665 nm (APC signal) and 615 nm (Europium signal) was used to evaluate the peptide recruitment on the receptors. The peptide sequences used were the following: biotin-CPSSHSSLTERHKILHRLLQEGSPS-COOH for SRC-1 (spanning aminoacids 676-700, reference number NP_671766), biotin-DGTPPPQEAEEPSLLKKLLLAPANT-COOH for PGC-1α (spanning aminoacids 130-154, reference number NP_037393), biotin-LERNNIKQAANNSLLLHLLKSQTIP-COOH for RIP140 (spanning aminoacids 366-390, reference number NP_003480), biotin-SGNLVPDAASKHKQLSELLRGGSGS-COOH for CBP/P300 (spanning aminoacids 56-80, reference number NP_004371), biotin-GSTHGTSLKEKHKILHRLLQDSSSPVD-COOH for TIF2 (spanning aminoacids 676-702, reference number NP_006531), biotin-PVSSMAGNTKNHPMLMNLLKDNPAQ-COOH for TRAP220 (spanning aminoacids 631-655, reference number NP_038662), and biotin-SFADPASNLGLEDIIRKALMGSFDD-COOH for NcoR (spanning aminoacids2253ï2277, reference number NP_006302). For competitive assays, the ERŬprotein was cleaved by thrombin to remove the histidine tag and added to a mix containing the previously described amount of LXRŬ to create 12-point dilution curves (starting from 28 ng of ERŬ). T0901317 at 30 nM (the experimentally determined EC50, data not shown) and the indicated coactivator at the EC50 were determined.

ChIP-qPCR: The primer sequences used in ChIP-qPCR analysis are the following:

**Gene**	**Forward**	**Reverse**
Abcg5	5´-CTCTGGACTCAGAAAGGGCA-3´	5´-TCAGTTAAAGCTGCCCTGGA-3´
Abca1	5´-AAACTGAGAGGGCAGGATGA-3´	5´-TGGAAGACTGTGAAGGCTGT-3´
Cyp7a1	5´-CCTTGAACTAAGTCCATCTTCTC-3´	5´-CCAGCTTTGAATGTTATGTCAG-3´
Cyp27a1	5´-AAGATGGATAGGGCAGAAAG-3´	5´-CTGGAACAGGACAGAATCAG-3´
Shp	5´-AGGGGCTCTGAATGATCTTC-3´	5´-GGGGACTTTGCTCACTGTAC-3´
Srebp-1c	5´-GATCAAAGCCAGACGCCGT-3´	5´-CATCCCCGAAAAGAGCCTG-3´
FoxL2	5´-GCTGGCAGAATAGCATCCG-3´	5´-TGATGAAGCACTCGTTGAGGC-3´

SUPPLEMENTAL REFERENCES

Adorni, M.P., Zimetti, F., Puntoni, M., Bigazzi, F., Sbrana, F., Minichilli, F., Bernini, F., Ronda, N., Favari, E., and Sampietro, T. (2012). Cellular cholesterol efflux and cholesterol loading capacity of serum: effects of LDL-apheresis. J Lipid Res *53*, 984-989.

Bates D, C.J., Dalgaard P, Falcon S, Gentleman R, et al. (2014). r-project.

Favari, E., Lee, M., Calabresi, L., Franceschini, G., Zimetti, F., Bernini, F., and Kovanen, P.T. (2004a). Depletion of pre-beta-high density lipoprotein by human chymase impairs ATP-binding cassette transporter A1- but not scavenger receptor class B type I-mediated lipid efflux to high density lipoprotein. J Biol Chem *279*, 9930-9936.

Favari, E., Ronda, N., Adorni, M.P., Zimetti, F., Salvi, P., Manfredini, M., Bernini, F., Borghi, C., and Cicero, A.F. (2013). ABCA1-dependent serum cholesterol efflux capacity inversely correlates with pulse wave velocity in healthy subjects. J Lipid Res *54*, 238-243.

Favari, E., Zanotti, I., Zimetti, F., Ronda, N., Bernini, F., and Rothblat, G.H. (2004b). Probucol inhibits ABCA1-mediated cellular lipid efflux. Arterioscler Thromb Vasc Biol *24*, 2345-2350.

Khera, A.V., Cuchel, M., de la Llera-Moya, M., Rodrigues, A., Burke, M.F., Jafri, K., French, B.C., Phillips, J.A., Mucksavage, M.L., Wilensky, R.L., *et al.* (2011). Cholesterol efflux capacity, high-density lipoprotein function, and atherosclerosis. N Engl J Med *364*, 127-135.

Pedrioli, P.G., Eng, J.K., Hubley, R., Vogelzang, M., Deutsch, E.W., Raught, B., Pratt, B., Nilsson, E., Angeletti, R.H., Apweiler, R., *et al.* (2004). A common open representation of mass spectrometry data and its application to proteomics research. Nat Biotechnol *22*, 1459-1466.

Rozowsky, J., Euskirchen, G., Auerbach, R.K., Zhang, Z.D., Gibson, T., Bjornson, R., Carriero, N., Snyder, M., and Gerstein, M.B. (2009). PeakSeq enables systematic scoring of ChIP-seq experiments relative to controls. Nat Biotechnol *27*, 66-75.

Smith, C.A., Want, E.J., O'Maille, G., Abagyan, R., and Siuzdak, G. (2006). XCMS: processing mass spectrometry data for metabolite profiling using nonlinear peak alignment, matching, and identification. Anal Chem *78*, 779-787.

Villa, A., Della Torre, S., Stell, A., Cook, J., Brown, M., and Maggi, A. (2012). Tetradian oscillation of estrogen receptor alpha is necessary to prevent liver lipid deposition. Proceedings of the National Academy of Sciences of the United States of America *109*, 11806-11811.

Zimetti, F., Weibel, G.K., Duong, M., and Rothblat, G.H. (2006). Measurement of cholesterol bidirectional flux between cells and lipoproteins. J Lipid Res *47*, 605-613.
